# Hydrogen-Bonded Interfacial Super-Assembly of Spherical Carbon Superstructures for High-Performance Zinc Hybrid Capacitors

**DOI:** 10.1007/s40820-025-01883-1

**Published:** 2025-08-25

**Authors:** Yang Qin, Chengmin Hu, Qi Huang, Yaokang Lv, Ziyang Song, Lihua Gan, Mingxian Liu

**Affiliations:** 1 Shanghai Key Lab of Chemical Assessment and Sustainability, School of Chemical Science and Engineering, Tongji University, 1239 Siping Rd., Shanghai, 200092 People’s Republic of China; 2 State Key Laboratory of Pollution Control and Resource Reuse, College of Environmental Science and Engineering, Advanced Research Institute, Tongji University, 1239 Siping Rd., Shanghai, 200092 People’s Republic of China; 3 Department of Chemistry, Shanghai Key Lab of Molecular Catalysis and Innovative Materials and Collaborative Innovation Center of Chemistry for Energy Materials, Fudan University, 2005 Songhu Rd., Shanghai, 200438 People’s Republic of China; 4 Institute for Electric Light Sources, School of Information Science and Technology, Fudan University, 2005 Songhu Rd., Shanghai, 200438 People’s Republic of China; 5 College of Chemical Engineering, Zhejiang University of Technology, 18 Chaowang Rd., Hangzhou, 310014 People’s Republic of China; 6 State Key Laboratory of Cardiovascular Diseases and Medical Innovation Center, Shanghai East Hospital, School of Medicine, Tongji University, 150 Jimo Rd., Shanghai, 200120 People’s Republic of China

**Keywords:** Hydrogen bonds, Interfacial super-assembly, Spherical carbon superstructures, Zn hybrid capacitors, Energy storage

## Abstract

Carbon superstructures with multiscale hierarchies and functional attributes represent an appealing cathode candidate for zinc hybrid capacitors, but their tailor-made design to optimize the capacitive activity remains a confusing topic. Here we develop a hydrogen-bond-oriented interfacial super-assembly strategy to custom-tailor nanosheet-intertwined spherical carbon superstructures (SCSs) for Zn-ion storage with double-high capacitive activity and durability. Tetrachlorobenzoquinone (H-bond acceptor) and dimethylbenzidine (H-bond donator) can interact to form organic nanosheet modules, which are sequentially assembled, orientally compacted and densified into well-orchestrated superstructures through multiple H-bonds (N–H···O). Featured with rich surface-active heterodiatomic motifs, more exposed nanoporous channels, and successive charge migration paths, SCSs cathode promises high accessibility of built-in zincophilic sites and rapid ion diffusion with low energy barriers (3.3 Ω s^−0.5^). Consequently, the assembled Zn||SCSs capacitor harvests all-round improvement in Zn-ion storage metrics, including high energy density (166 Wh kg^−1^), high-rate performance (172 mAh g^−1^ at 20 A g^−1^), and long-lasting cycling lifespan (95.5% capacity retention after 500,000 cycles). An opposite charge-carrier storage mechanism is rationalized for SCSs cathode to maximize spatial capacitive charge storage, involving high-kinetics physical Zn^2+^/CF_3_SO_3_^−^ adsorption and chemical Zn^2+^ redox with carbonyl/pyridine groups. This work gives insights into H-bond-guided interfacial super-assembly design of superstructural carbons toward advanced energy storage.

**Supplementary Information:**

The online version contains supplementary material available at 10.1007/s40820-025-01883-1.

## Highlights

- The spherical carbon superstructures (SCS-6) are synthesized by a hydrogen-bonded interfacial super-assembly, owning surface-opening pores, interconnected channels and rich heteroatom species.
- Maximized accessibility of surface-active sites and opposite charge-carrier storage mechanism ensure high ion storage efficiency.
- The assembled zinc-ion hybrid capacitor based on SCS-6 delivers ultrahigh energy density (166 Wh kg^−1^) and super-stable cycle lifespan (500,000 cycles).

**Supplementary Information:**

The online version contains supplementary material available at 10.1007/s40820-025-01883-1.

## Graphical abstract

**Figure:**
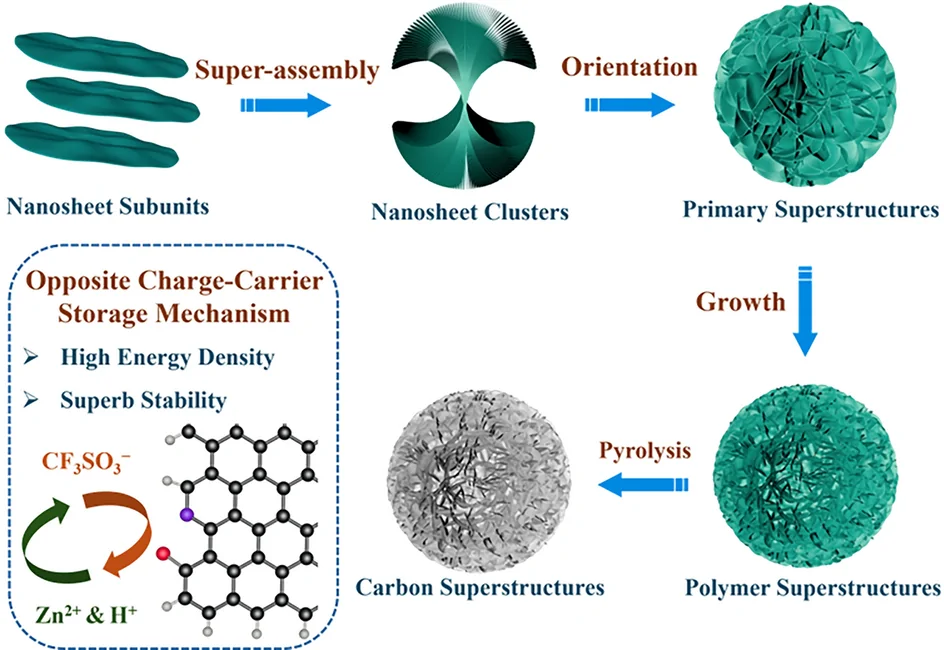

## Introduction

Aqueous zinc hybrid capacitors (ZHCs), a newly emerged energy storage device, have garnered much attention due to their high energy and power output, long cycle life, excellent safety, and environmental friendliness [1, 2]. A ZHC combines battery-type Zn anode and capacitor-type cathode. Zn anode supplies high capacity through Zn deposition and stripping reaction, while the cathode delivers fast power output through Zn-ion adsorption and desorption [3–6]. Given the high theoretical capacity of 820 mAh g^−1^ and the low redox potential of - 0.76 V (vs. the standard hydrogen electrode) of Zn anodes, capacitive carbon cathodes have been widely studied for pairing with a Zn anode to boost Zn^2+^ storage [7–9]. In comparison to the use of redox-active molecules as electrolyte additives [10–13], the advantages of carbons include low cost, high conductivity, and tunable nanostructures. Porous carbon materials still face challenges related to poor surface accessibility and sluggish ion kinetics [14–19], due to inevitable stacking and aggregation of low-dimensional (0-2D) carbon materials or the lack of interconnected channels in 3D carbon structures [20–27]. As a result, designing well-arranged porous carbon nanostructures becomes a crucial objective for improving the performance of ZHCs.

Integrating low-dimensional building blocks (e.g., nanosheets, nanoparticles) into well-organized superstructures offers a promising approach to achieve innovative multiscale hierarchies with enhanced functional properties, making them highly valuable for electrochemical energy storage applications [28–32]. Construction of 3D carbon superstructures (CSs) is a feasible method to maximize the surface accessibility and improve ion transport efficiency in carbon-based cathodes [33–37]. CSs are constructed from the assembly of low-dimensional building blocks such as 0D quantum dots, 1D carbon nanotubes, or 2D graphene sheets [38–42]. These structures not only retain the unique properties of their individual components but also gain specific advantages from their overall 3D architecture making them highly suitable for energy storage [43–47]. For example, 3D flower-like carbon superstructures composed of two-dimensional mesoporous nanosheet modules provide abundant active sites and a highly conductive, interconnected network for efficient charge storage [48, 49]. However, current methods for synthesizing CSs face challenges related to complex reaction steps and structural uncontrollability [50–53]. In most cases, low-dimensional building blocks, such as 1D carbon nanotubes or 2D graphene oxides, must be pre-synthesized prior to form high-dimensional CSs [54–58]. Additionally, CSs often exhibit irregular configurations because polymeric soft matter precursors tend to aggregate [59]. Organic polymeric subunit-based hierarchical superstructures are rarely reported for the design of CSs due to the inherent thermodynamic instability of polymeric materials [60]. Thus, it remains a significant challenge to precisely manipulate super-assembled building blocks for achieving fine-tunable CSs cathodes toward better ZHCs.

In this study, we present a hydrogen-bond-oriented interfacial super-assembly approach to fabricate spherical carbon superstructures (SCSs) tailored for enhanced Zn-ion storage with high capacitive activity and exceptional durability. Tetrachlorobenzoquinone, as a hydrogen-bond acceptor, and dimethylbenzidine, as a donor, interact to form organic nanosheets. These nanosheets are sequentially assembled, orientally compacted, and densified into well-ordered superstructures via multiple hydrogen bonds (N–H···O). The resulting SCSs exhibit surface-active heterodiatomic motifs, open nanoporous channels, and continuous charge migration pathways, ensuring high accessibility to zincophilic sites and rapid ion diffusion with minimal energy barriers. As a result, Zn||SCSs capacitor achieves high energy density, high-rate capability, and an impressive cycling lifespan. An opposite charge-carrier storage mechanism is proposed for SCSs cathode, combining high-kinetics physical adsorption of Zn^2^⁺/CF_3_SO_3_⁻ ions and chemical Zn^2+^ redox with carbonyl/pyridine groups. This study provides valuable insights into hydrogen-bond-guided superstructural carbon design for advanced energy storage systems.

## Experimental Section

### Materials Synthesis

Firstly, 10 mmol of tetrachlorobenzoquinone (TBQ) is dissolved in 50 mL dimethylsulfoxide (DMSO), *N*,*N*-dimethylformamide (DMF), or dimethylacetamide (DMAc) to form a TBQ solution; also 5 mmol of dimethylbenzidine (DMB) is dissolved in 50 mL DMSO, DMF, or DMAc forming DMB solution. Secondly, DMB solution is dropped into TBQ solution contained in round flask at room temperature and keep stirring vigorously for 30 min. Thirdly, the mixed solution is transferred to Teflon reactor and kept at 80 °C for certain time (0.5, 2, 4, and 6 h) to promote polymerization. The polymer products are obtained after filtration by washing with DMSO, water, and ethanol, respectively. The resultant polymers are marked with SPS-*x*, where *x* refers to polymerization reaction time. To obtain porous carbons, the polymers are mixed with NaNH_2_ serving as an activator (a mass ratio of 1:1). Then the mixture is heated to 800 °C at a heating rate of 3 °C min^−1^ under N_2_ atmosphere and then kept for 2 h to complete the activation. The resultant porous carbon materials are marked with SCS-*x*, where *x* refers to reaction time.

### Characterization

The morphology is analyzed by scanning electron microscope (SEM, JSM-7900F) and transmission electron microscopy (TEM, JEM-2100). The surface element composition and state of the products are tested via X-ray photoelectron spectrometer (XPS, AXIS Ultra DLD). N_2_ adsorption and desorption isotherms are tested on a Micromeritics of ASAP 2460 apparatus at –196 °C. The specific surface area and pore size distributions are calculated by the Brunauer–Emmett–Teller model within *P*/*P*_0_ = 0.05–0.25 and density functional theory equilibrium model, respectively. Raman spectroscopy (Renishaw Invia, the laser excitation *λ* = 514 nm) is used to analyze graphitization degree of carbon materials, and X-ray powder diffraction technique (XRD) is utilized to test crystalline structure of products.

### Electrochemical Measurements

The working electrodes consist of resultant porous carbon (SCS-*x*), graphite, and polytetrafluoroethylene (PTFE) binder (8:1:1 mass ratio). The mass loading of active materials in cathodes is ~ 5 mg cm^−2^. The above mixture is soaked in ethanol and dispersed by ultrasound for 2 h and then dried in oven at 100 °C for 12 h. Next, the dried mixture is pressed on stainless steel mesh. Zn foil is used as an anode, the working electrode is used as a cathode, 3 mol L^−1^ Zn(CF_3_SO_3_)_2_ aqueous solution is used as an electrolyte, and a glassy fibrous is used as separator in zinc ion hybrid capacitors (ZIHCs).

Galvanostatic charging/discharging (GCD) tests are carried out on the CT3001A battery test system with a potential in a range of 0–1.8 V. Cyclic voltammetry (CV) and electrochemical impedance spectroscopy (EIS) are investigated through CHI660E electrochemical workstation. The gravimetric capacitance (*C*_m_, F g^−1^) of the working electrode is calculated by Eq. (1):

1 $$ C_{{\mathrm{m}}} = \frac{I \times \Delta t}{m} $$

where *I* refers to the current density, Δ*t* represents the discharging time, and *m* is the mass of HCS-*x* sample. Energy density (*E*) and power density (*P*) are calculated by Eqs. (2) and (3):

2 $$ E = C_{{\mathrm{m}}} \times \Delta V $$

3 $$ P = \frac{{C_{{\mathrm{m}}} \times \Delta V}}{1000 \times \Delta t} $$

where Δ*V* is the voltage window.

The ion diffusion coefficient (*D*, cm^2^ s^−1^) is calculated by Eq. (4):

4 $$ D = \frac{{R^{2} T^{2} }}{{2A^{2} C^{2} F^{4} n^{4} \sigma^{2} }} $$

where *R* (8.314 J mol^−1^ K^−1^) is gas constant, *T* (293.15 K) is Kelvin temperature, *A* (m^2^ g^−1^) is the area of the electrode surface, *c* (mol L^−1^) is molar concentration of the electrolyte, *n* is Faraday constant, and *σ* (Ω s^−0.5^) is diffusive resistance.

The capacitance *C*(*ω*) changes along with the frequency which is defined as follows:

5 $$ C\left( \omega \right) = C^{\prime}\left( \omega \right) - jC^{\prime\prime}\left( \omega \right) $$

where *C*′(*ω*) is the real part of *C*(*ω*), the low frequency value of *C*′(*ω*) refers to the capacitance of the device tested in constant-current discharge process, and *C*″(*ω*) is the imaginary part associated with the energy dissipation by an irreversible process leading to a hysteresis. *ω* is the angular frequency given by *ω* = 2*πf*.

The relaxation time constant (*τ*_0_) is calculated by Eq. (6):

6 $$ \tau_{0} = {1}/f_{0} $$

*τ*_0_ is marked at the position where the imaginary part of the capacitance (*C*″) reaches its maximum at frequency *f*_0_.

### Activation Energy

The activation energy (*E*_a_, kJ mol^−1^) for the charge transfer process can be deduced using the Arrhenius equation:

7 $$ R_{{{\mathrm{ct}}}}^{{ - {1}}} = A{\mathrm{exp}}\left( { - E_{{\mathrm{a}}} /{\mathrm{RT}}} \right) $$

where *R*_ct_ is the charge transfer resistance (Ω), *A* is constant under a stable experimental condition,* R* represents the gas constant (8.314 J mol^−1^ K^−1^), and *T* is the temperature (*T*). The ln(*R*_ct_^−1^) values were plotted vs. 1000/*T*, and linear fitting was carried out to collect *E*_a_:

8 $$ {\mathrm{ln}}\left( {R_{{{\mathrm{ct}}}}^{{ - {1}}} } \right) = - E_{{\mathrm{a}}} /RT + k $$

where *k* is constant.

### Optical Energy Gap

The optical energy gaps (*E*_g_, eV) of carbon cathodes can be determined by the ultraviolet–visible (UV–Vis) spectra, which is expressed as:

9 $$ \alpha \frac{{(hv - E_{{\mathrm{g}}} )^{1/2} }}{hv} $$

10 $$ hv = { 128}0/\lambda $$

where *α* denotes the optical absorption coefficient, *hv* is the photon energy, *λ* is the wavelength.

### Theoretical Calculations

Reduce density gradient (RDG) analysis: theoretical calculations were performed via the Gaussian 16 suite of programs. The structures of the studied compound (M) and its complexes were fully optimized at the B3LYP-D3/def2-SVP level of theory. The vibrational frequencies of the optimized structures were carried out at the same level. The structures were characterized as a local energy minimum on the potential energy surface by verifying that all the vibrational frequencies were real. The interaction energy of the optimized complex was also calculated. The reduce density gradient (RDG) analysis was performed with Multiwfn program [61, 62], where RDG value provides interaction strength, while sign(*λ*_2_)*ρ* value shows interaction types. The Visual Molecular Dynamics (VMD) program was used to plot the RDG color-filled isosurface maps [63]. The surfaces are displayed on a blue–green–red scale with respect to the values of sign(*λ*_2_)*ρ*, ranging from − 0.04 to 0.02 a.u.

The charge density difference (Δ*ρ*) of Zn atom and carbon framework in adsorption process is calculated to analyze the bonding nature of Zn^2+^ adsorbed on the dual doped carbon framework and charge transfer property. The charge transfer level of Zn^2+^ and O/N-substituted active sites is calculated by a Bader charge analysis pattern.

11 $$ \Delta \rho = \rho_{{{\mathrm{H}}/{\mathrm{carbon}}}} {-}\rho_{{{\mathrm{carbon}}}} {-}\rho_{{\mathrm{H}}} $$

where *ρ*_H/carbon_ is total charge density, *ρ*_carbon_ is carbon matrix part charge density, and *ρ*_H_ is adsorbent charge density.

## Results and Discussion

### Fabrication and Morphologies of Spherical Carbon Superstructures

**Fig. 1:**
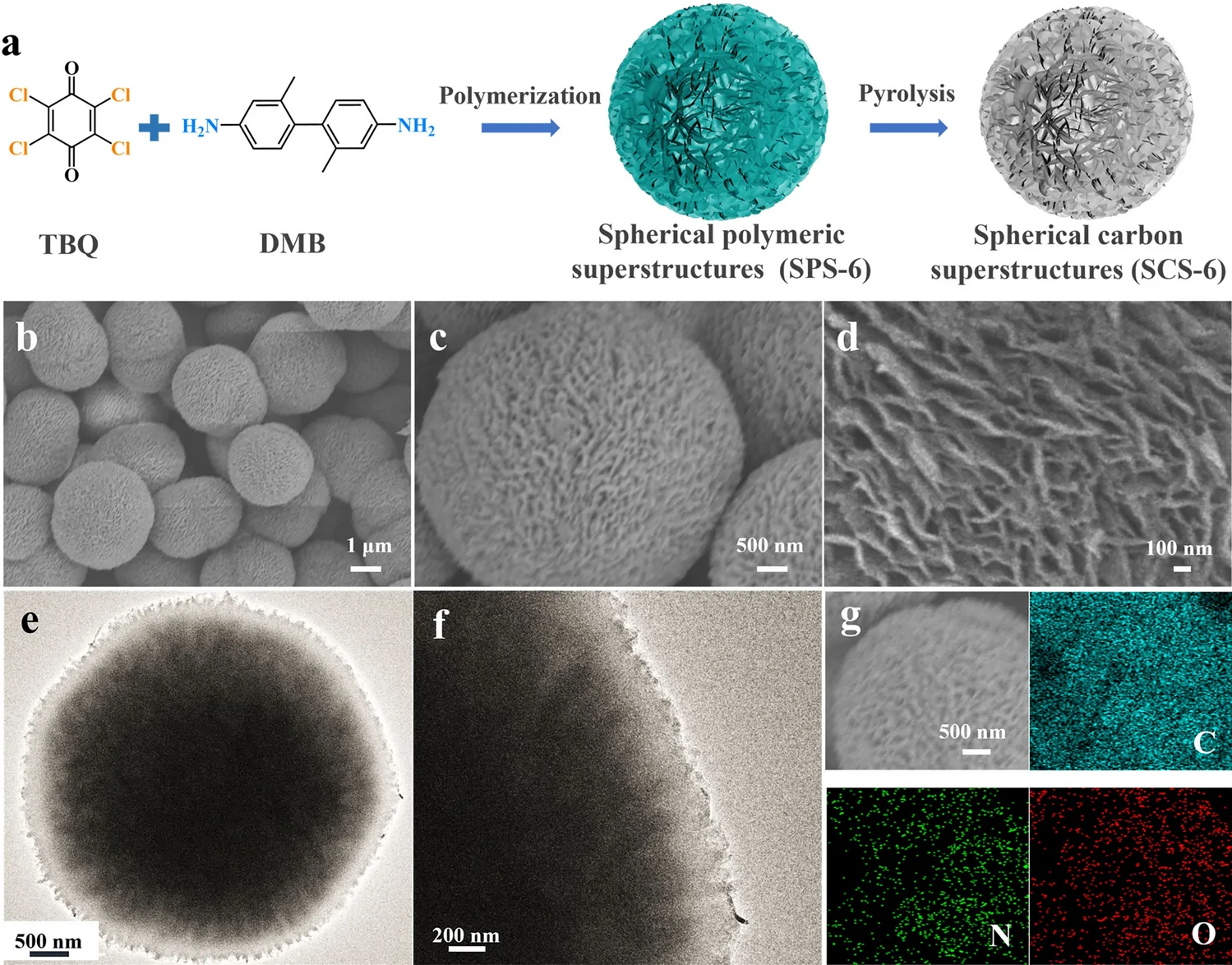
Fabrication and morphologies of spherical carbon superstructures.** a** Schematic diagram for the formation of SCS-6. **b–d** SEM images. **e–f** TEM images. **g** element maps of SCS-6

Figure 1a presents a schematic illustration of typical spherical carbon superstructures (SCS-6). The synthesis follows two main steps: (1) spherical polymeric superstructures (SPS-6) are first assembled via a nucleophilic aromatic substitution reaction between the –Cl groups of tetrachlorobenzoquinone (TBQ) and the –NH_2_ groups of dimethylbenzidine (DMB). This reaction enables the interfacial self-assembly of 2D nanosheets into 3D spherical superstructures; (2) SCS-6 with retained superstructure frameworks is subsequently obtained through pyrolysis. During the high-temperature pyrolysis process, NaNH_2_ can decompose into NH_3_ and NaOH, where NH_3_ compensates extra N heteroatoms and NaOH etches the polymeric skeleton to form porous structures. According to SEM images (Fig. 1b, c), SCS-6 presents micro-scaled monodisperse 3D spherical morphologies. High-magnification SEM image (Fig. 1d) reveals that the surface consists of closely stacked 2D nanosheets, creating surface open pores due to the inherent spacing between the layers. Additionally, SCS-6 shows a lose inner layer with abundant interconnected pores (Fig. 1e, f and S1).

Particle size analysis using dynamic light scattering characterization shows that SCS-6 processes a narrow size distribution, ranging between 3  and  4.2 μm, with an average size of 3.6 μm (Fig. S2). After subjecting SCS-6 to a 6-h ultrasonic process, nanosheets approximately 40 nm in thickness are observed (Fig. S3), confirming that the 3D structure is indeed composed of nanosheet modules. This ultrasonic treatment likely aids in exfoliating the stacked nanosheets. Elemental maps show that C, N, and O elements are homogeneously distributed on SCS-6 (Fig. 1g). During the H-bond-oriented interfacial super-assembly process, the whole polymeric system tends to form larger particles with lower surface energy, which is known as Ostwald ripening. Through this effect, 2D nanosheets will adhere nearby, achieving the interfacial self-assembly to form cross-linked 3D spherical superstructures. Collectively, SCS-6 possesses 3D monodispersed spherical morphologies with surface-opening pores, interconnected channels, and rich heteroatom species.

### Super-Assembly Mechanism of Spherical Carbon Superstructures

**Fig. 2:**
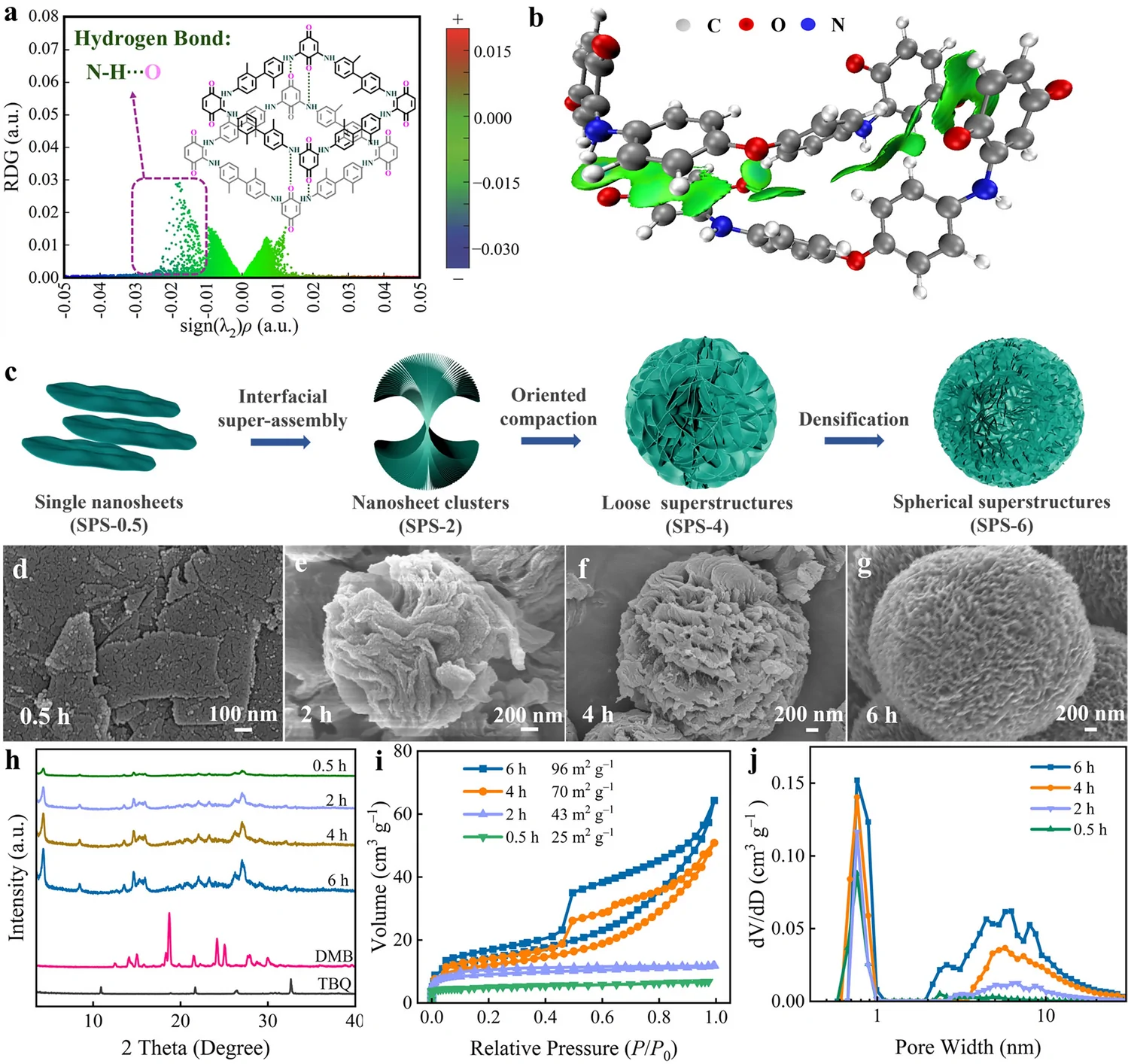
Super-assembly mechanism of spherical carbon superstructures. **a** Plots of RDG *vs*. sign(*λ*_2_)*ρ* and **b** corresponding isosurfaces. **c** Schematic demonstration of module interfacial super-assembly. **d–g** SEM images of polymeric products at different times of 0.5, 2, 4, and 6 h. **h** XRD patterns of TBQ, DMB, and polymeric products. **i** Nitrogen absorption/desorption isotherms. **j** Pore-size distribution curves of polymeric superstructures at different polymerization times

To better understand the super-assembly mechanism of 2D nanosheet modules, a combination of theoretical simulations and ex situ characterizations was conducted to observe the dynamic formation process of SPS. Polymeric products synthesized at different reaction times were denoted as SPS-*x*, where *x* indicates the reaction time in hours (e.g., SPS-0.5, SPS-2, SPS-4, and SPS-6). To identify the driving force of the super-assembly process, we employed reduced density gradient (RDG) analysis, which is a powerful tool for detecting both covalent and non-covalent interactions. The repeating units of polymeric superstructures are served as the object for theoretical calculation (Fig. 2a, b). Notably, the RDG plots (Fig. 2a) reveal abundant hydrogen–bond interactions (N–H···O) among these repeating polymeric units, with the peak intensity in the hydrogen–bond region indicating strong non-covalent bonding that suggests hydrogen bonds are the primary driver of interfacial super-assembly.

Moreover, the morphologies of the polymeric products over time (Fig. 2c–g) illustrate the sequential development of the super-assembly, ultimately forming 3D spherical polymeric superstructures. Polymeric nanosheets (SPS-0.5, Figs. 2d and S4), nanosheet clusters (SPS-2, Fig. 2e), loose superstructures (SPS-4, Fig. 2f), and spherical polymeric superstructures (SPS-6, Fig. 2g) emerge chronologically in the process of interfacial super-assembly. This progression highlighted a chronological process of interfacial super-assembly: polymeric nanosheets (HPS-0.5) form initial through the nucleation and growth of oligomers, acting as building blocks of hierarchical polymeric superstructures (SPS-6). Through interfacial interaction, these nanosheets assemble into clusters (SPS-2), which are then driven by hydrogen bonds to undergo compaction, forming loosely packed superstructures (SPS-4). As hydrogen bonding continues to drive the process, these loosely packed structures densify, resulting in regular and tightly packed hierarchical arrangements rather than irregular dispersion [64]. The entire super-assembly process, propelled by hydrogen bonding forces, demonstrates how 2D polymeric nanosheets function as foundational modules for constructing 3D hierarchical polymeric superstructures. Using this 2D module-oriented super-assembly approach, we successfully synthesized polymeric superstructures in different solvents, including *N*,* N*-dimethylformamide (DMF) and dimethylacetamide (DMAc), with the same monomers. Interestingly, owing to the different interactions between the polymeric precursor and the solvent [42], the formed nanosheet building blocks undergo different super-assembly processes to form various geometries in DMF and DMAc solvents. Superstructures synthesized in DMF exhibit compact, curly flake morphologies (Fig. S5a, b), while those synthesized in DMAc show petal-like morphology, resembling blooming flowers (Fig. S5c, d).

Ex situ FT-IR and XRD analyses were performed to confirm the presence of interfacial hydrogen bonds among nanosheets during the super-assembly process. FT-IR spectra reveal the synthesis route of SPS (Fig. S6). In FT-IR spectra, the –C=O stretch band of the TBQ appears at 1620 cm⁻^1^; however, in the polymeric superstructures, it shifts to 1574 cm⁻^1^ (Fig. S7). It indicates the formation of H-bonds between polymeric nanosheets, attributed to strong interactions between –NH– and –C=O groups, which facilitate the interfacial super-assembly. Additionally, the XRD patterns of the polymeric superstructures displayed significant difference from those of TBQ and DMB monomers, indicating the emergence of a new crystalline structure resulting from the super-assembly process. The peak intensity at 27°, associated with hydrogen bonds, increases with longer polymerization times (Fig. 2h), further demonstrating the progression of interfacial super-assembly among the polymeric nanosheets. These findings collectively confirm the crucial role of H-bonding interactions in the interfacial super-assembly of polymeric nanosheets, leading to the formation of superstructures.

The gradual construction of porous structures within polymeric superstructures through the super-assembly process is confirmed by N_2_ adsorption–desorption analysis and pore size distribution curves (Fig. 2i, j). N_2_ adsorption and desorption curves of SCS-*x* exhibit combined I/IV-type isotherms. The specific surface area (SSA) of the polymeric products increased progressively with polymerization time, reaching values of 25, 43, 70, and 96 m^2^ g^−1^ for SPS-0.5, SPS-2, SPS-4, and SPS-6, respectively. The micropores in SPS-0.5, SPS-2, SPS-4, and SPS-6 are predominantly concentrated around 0.8 nm. Mesopores, ranging from 2 to 12 nm, were observed in SPS-2, SPS-4, and SPS-6, whereas HPS-0.5 exhibited very few mesopores. Generally, both the volume of meso- and micropores and the SSA increase with extended polymerization times. Due to the significant stacking and aggregation of polymeric nanosheets, SPS-0.5 exhibits the lowest SSA and few mesopores. Firstly, the interfacial super-assembly of nanosheets effectively prevents excessive stacking and aggregation. Secondly, the nanosheets assembled into a 3D structure led to the formation of additional surface-opening pores and interconnected mesopores. As a result, the SSA and the mesopores sequentially increases in SPS-2, SPS-4 and SPS-6.

### Physical structure of Spherical Carbon Superstructures

**Fig. 3:**
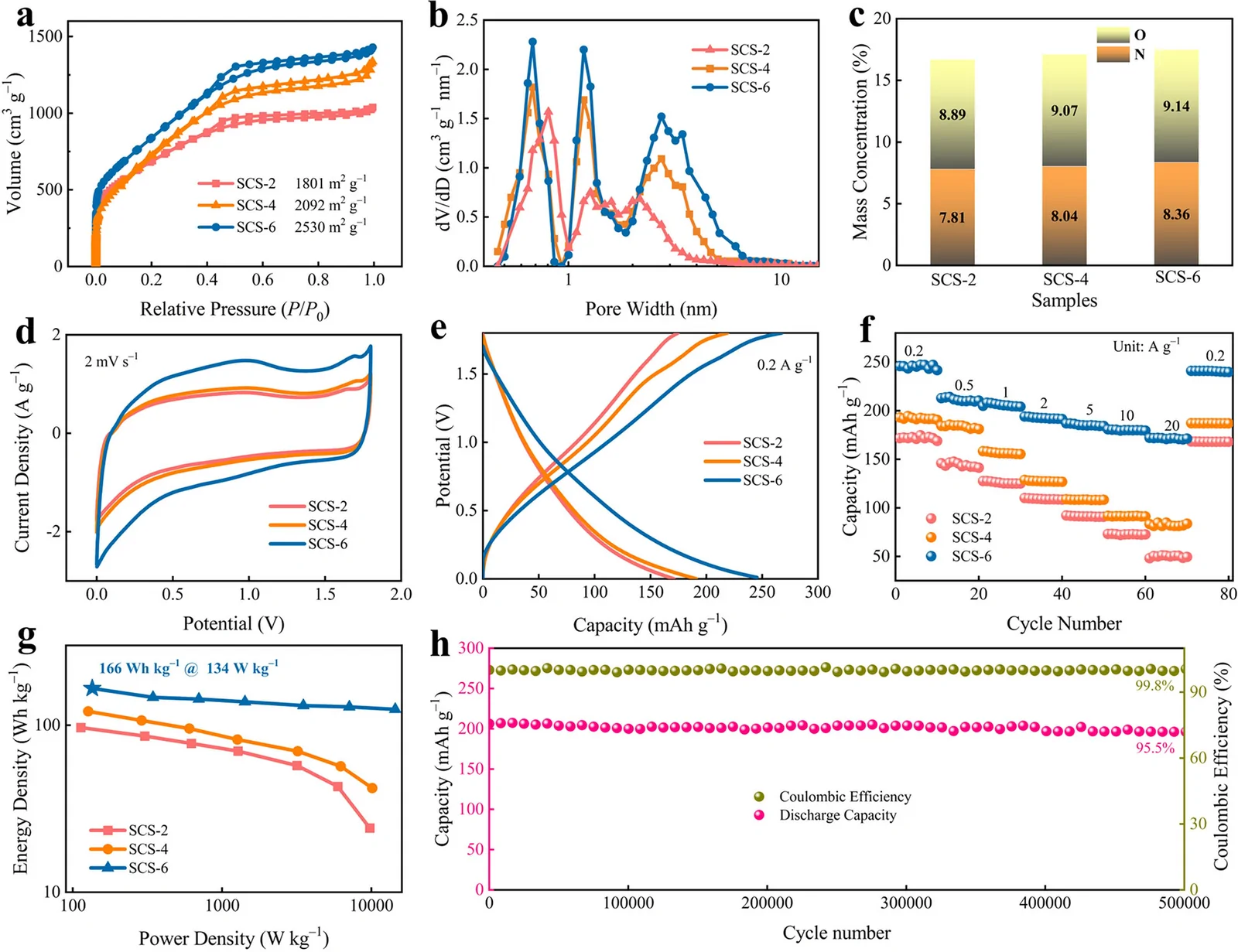
Physical structure and electrochemical performance of carbon superstructures*.* **a** Nitrogen absorption/desorption isotherms of SCS-*x*. **b** Pore-size distribution curves. **c** C, N, O contents obtained by XPS analysis. **d** CV curves at 2 mV s^–1^. **e** GCD curves at 0.2 A g^–1^. **f** Capacity reversibility of SCS-*x* cathodes from 0.2 to 20 A g^–1^. **g** Ragone plots. **h** Cyclability and coulombic efficiency of Zn||SCS-6 device

The polymeric superstructures (SPS-2, SPS-4, and SPS-6) are selected as precursors to synthesize corresponding hierarchical porous carbon superstructures (SCS-2, SCS-4, and SCS-6) at 800 °C (Fig. S8) for subsequent characterization and electrochemical evaluation. Nitrogen adsorption–desorption isotherms exhibit all exhibit type II isotherms and multiscale pore structure, comprising macropores, mesopores, and micropores of these samples (Fig. 3a), as confirmed by the steep adsorption at low relative pressure (*P*/*P*_0_ < 0.1), the capillary condensation step with faint hysteresis loops at 0.45 < *P*/*P*_0_ < 0.95, and a slight increase at *P*/*P*_0_ > 0.95. Among these samples, SCS-6 surpassed the others, exhibiting the highest specific surface area (SSA) of 2530 m^2^ g^−1^ and a total pore volume (*V*_total_) of 2.2 cm^3^ g^−1^, compared to SCS-4 (2092 m^2^ g^−1^, 1.6 cm^3^ g^−1^) and SCS-2 (1801 m^2^ g^−1^, 1.1 cm^3^ g^−1^) (Table S1). It is worth mentioning that all SCS-*x* samples exhibit significant mesoporosity, with SCS-6 showing a superior mesoporous volume ratio (*V*_meso_/*V*_total_) and mesoporous SSA (*S*_meso_) compared to SCS-2 and SCS-4. SCS-6 has 70% of *V*_meso_/*V*_total_, meanwhile SCS-4 has 53% and SCS-2 has 40%. The *S*_meso_ of SCS-6 has priority at 1047 m^2^ g^−1^, compared to 248 m^2^ g^−1^ of SCS-2 and 484 m^2^ g^−1^ of SCS-4. Moreover, according to the pore size distribution, the mesopores in SCS-2, SCS-4, and SCS-6 are predominantly distributed between 2–10 nm, while the micropores are centered at approximately 0.8 and 1.2 nm (Fig. 3b). The intensity of the peaks corresponding to mesopores and micropores increases sequentially from SCS-2 to SCS-6. Overall, the total porosity and mesoporosity of SCS-*x* samples progressively improve with the interfacial super-assembly process of the nanosheets. Among them, SCS-6 demonstrates outstanding performance with superior SSA, *V*_total_, *V*_meso_/*V*_total_, and *S*_meso_, reflecting an integrated structural advantage, where 2D nanosheet building blocks contribute abundant accessible surface area, while super-assembled 3D superstructures prevent nanosheet stacking and create interconnected mesoporous channels.

According to X-ray photoelectron spectroscopy (XPS) analysis, SCS-*x* samples consist of carbon (C 1*s* at 284.8 eV), nitrogen (N 1*s* at 400.7 eV), and oxygen (O 1*s* at 532.9 eV) elements (Fig. S9). The O content of SCS-*x* samples is 8.89 wt%, 9.07 wt%, and 9.14 wt% and the N content is 7.81 wt%, 8.04 wt%, and 8.36 wt%, corresponding to SCS-2, SCS-4, and SCS-6 (Fig. 3c). The similar N and O contents have arisen from the shared precursors and identical pyrolysis conditions used for all samples. For the O 1*s* species, three distinct peaks are observed: O–N (530.5 eV), C–O (532.0 eV), and C= O (533.4 eV) (Fig. S10) [65]. Similarly, the N 1*s* spectrum displays peaks corresponding to pyrrolic nitrogen (N-5 at 399.8 eV), pyridinic nitrogen (N-6 at 398.3 eV), and quaternary nitrogen (N-Q at 401.2 eV) (Fig. S11) [66, 67]. N/O doping in SCS-6 cathode can upgrade the surface wettability to enable high ion accessibility of electroactive sites and trigger faradaic features to undergo reversible redox reactions for unlocking superior capacitive activity and durability. Additionally, Raman spectra of SCS-*x* samples show two characteristic peaks: the *D* band at 1348 cm^–1^, associated with disordered carbon, and the *G* band at 1583 cm^–1^, corresponding to graphitic carbon. The *I*_D_/*I*_G_ ratio, which indicates degree of graphitization, ranges from 0.84 to 0.86 for SCS-*x* samples, suggesting the presence of graphite microcrystals (Fig. S12a). Furthermore, X-ray diffraction (XRD) patterns displayed peaks corresponding to the (002) and (100) diffraction planes, indicating partial graphitization and structural disorder within SCS-*x* materials (Fig. S12b).

### Electrochemical Performance of Spherical Carbon Superstructures

The electrochemical performance of SCS-*x* as cathodes in ZHCs is systematically investigated. The graphite conductive agent shows an extremely low specific capacity of 7 mAh g^−1^ at 0.2 A g^−1^ (Fig. S13), indicating its ignorable contribution to the total charge storage of SCS-6 cathode. A quasi-rectangular cyclic voltammetry (CV) profiles are observed with redox signals at 2 mV s^−1^, located at 0.9/1.1 V. CV curves suggest the combined electric double-layer capacitance and pseudocapacitive processes, which are attributed to electrochemically active O/N sites on SCS-*x* (Fig. 3d). The area of CV profiles corresponds to the specific capacity of SCS-*x* cathodes, which is quantitatively confirmed by galvanostatic charge/discharge (GCD) curves (Figs. 3e and S14). The specific capacities of SCS-2, SCS-4, SCS-6, and SCS-8 are 171, 191, 246, and 242 mAh g^−1^ at 0.2 A g^−1^ (Fig. S15), respectively. Detailed GCD curves at various current densities and CV curves at different scan rates are provided in Fig. S16. Figure 3f shows the capacity of SCS-*x* electrodes over continuous ten cycles at 0.2–20 A g^−1^. Remarkably, the devices regain their original capacities when returning to 0.2 A g^−1^ after cycling. The capacity retention from 0.2 to 20 A g^−1^ is 29% for SCS-2, 43% for SCS-4, and 70% for SCS-6 (Fig. S17).

In contrast to SCS-2 and SCS-4 with undeveloped superstructures, SCS-6 has the highest surface area to expose more active sites and highly open channels (Fig. 3a, b) to activate high-kinetics ion diffusion, for unlocking superior rate capacity (Fig. 3f). Of note, the more exposed inner active sites of SCS-6 cathode are easily accessible to electrolyte ions with relatively low energy barriers at low scan rates, thus affording high capacity. While they are inevitably blocked at increased scan rates, which lead to difficulty for ionic carriers with high energy barriers to utilize the active sites buried in the cathode, consequently resulting in a sudden drop in capacity.

So far, SCS-6 exhibits the highest specific capacity and the best rate capability, attributed to its ultrahigh SSA providing a large adsorption platform and its abundant mesopores enabling rapid ion transport. The Ragone plots in Fig. 3g reveal that SCS-6-based ZHCs deliver an ultrahigh energy density of 166 Wh kg^−1^ at 134 W kg^−1^, maintaining 124 Wh kg^−1^ at 14.5 kW kg^−1^. In comparison, SCS-4 offers 121 Wh kg^−1^ at 127 W kg^−1^ and 42 Wh kg^−1^ at 10.1 kW kg^−1^, while SCS-2 provides 97 Wh kg^−1^ at 113 W kg^−1^ and 24 Wh kg^−1^ at 9.7 kW kg^−1^. The SCS-6 also demonstrates outstanding cycling stability, maintaining 95.5% capacity retention over 500,000 cycles at 20 A g^−1^ with a coulombic efficiency of 99.8% (Figs. 3h and S18). The superstructures of SCS-6 can be well-maintained after cycling (Fig. S19), indicating its morphological stability. The slight difference between the cycling capacity and the rate performance at 20 A g⁻^1^ is considered to be error, which may originate from batch-to-batch variations (slight differences in electrode homogeneity and active material loading) in the cathode during the electrochemical process. Of note, during the cycling process of Zn||CSC-6 capacitor, a thick glass fiber separator (50 µm) was used to alleviate the problem of dendrite growth in the Zn foil anode (with a thickness of 50 µm) to achieve stable performance even after 500,000 cycles. When compared to recently reported carbon-based ZHCs (Table S2), Zn||SCS-6 outperformed in terms of specific capacity and energy density. Zn||SCS-6 capacitors with high-mass-loading SCS-6 cathode (10.2 mg cm^−2^) shows a capacity of 230 mAh g^−1^ at 0.2 A g^−1^, together with 81.2% capacity retention over 50,000 cycles at 20 A g^−1^ (Fig. S20), indicating its practical prospect. Notably, the observed trend in specific capacities, rate capabilities, and energy densities correlated with the SSA, *V*_meso_/*V*_total_, and *S*_meso_ of the samples, underscoring the critical role of 2D subunits and rich mesopores in enhancing electrolyte accessibility and charge transport.

### Kinetics Analysis of Spherical Carbon Superstructures

**Fig. 4:**
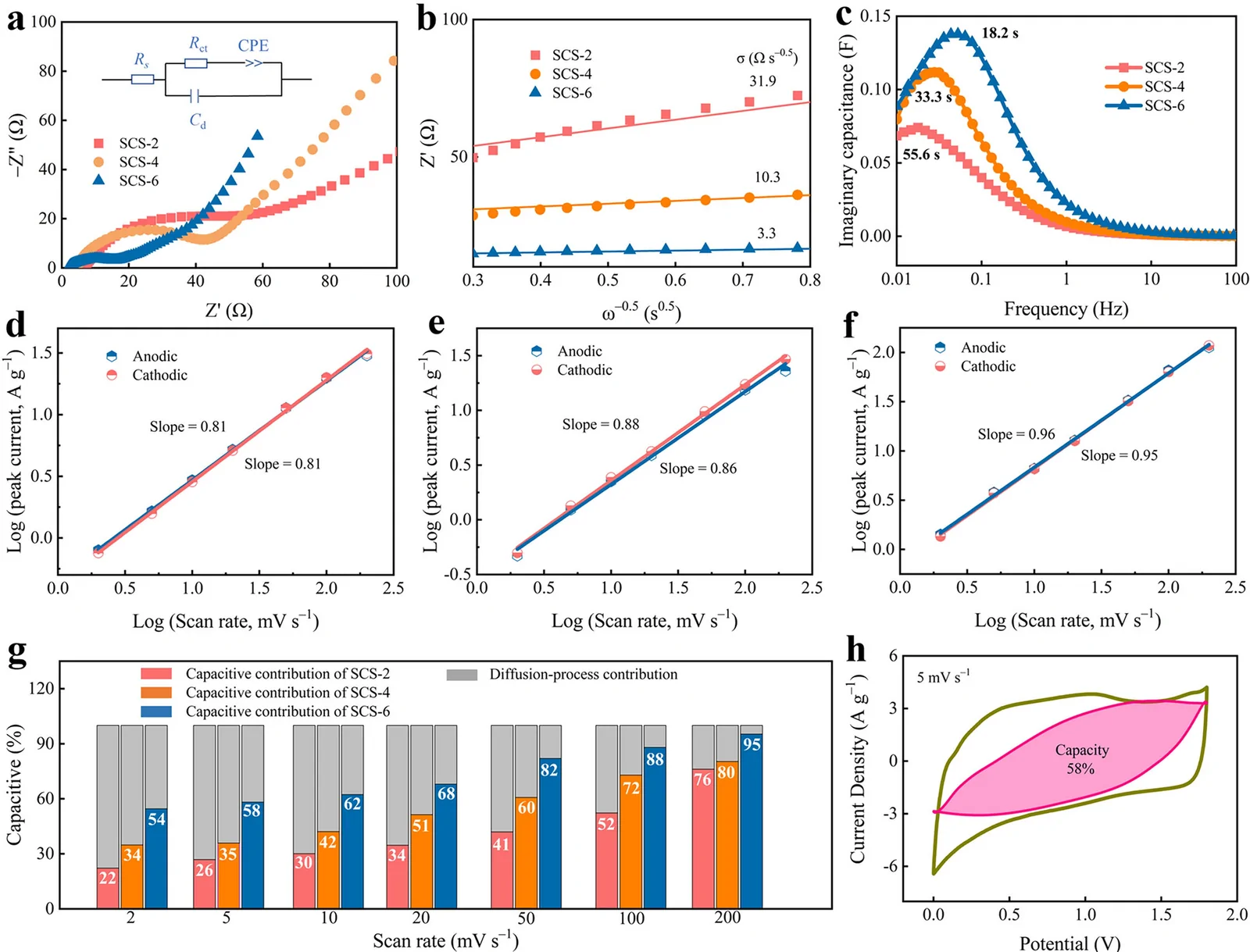
Investigation of electron/ion transfer behavior and charge storage kinetics of carbon superstructures. **a** Nyquist plots (inset: equivalent circuit). **b** diffusive resistance. **c** imaginary capacitance versus frequency. **d–f** *b* values for SCS-2, SCS-4, and SCS-6. **g** Ratios of capacitive and diffusion-controlled contribution. **h** Decoupling of the capacity contributed by capacitive (read shadow) and diffusion-controlled contribution of SCS-6

Electrochemical impedance spectroscopy (EIS) of SCS-*x* cathodes is analyzed to elucidate the ion diffusion kinetics within SCS-*x* cathodes. Nyquist plots display semicircles followed by linear tails (Fig. 4a), indicative of a combined kinetics and diffusion-controlled process [68]. SCS-*x* cathodes exhibit low equivalent series resistances (*R*_s_), with values of 7 Ω for SCS-2, 5.7 Ω for SCS-4, and 3.5 Ω for SCS-6. Similarly, the charge transfer resistance (*R*_ct_) decreases from 55.6 Ω for SCS-2 to 27.8 Ω for SCS-4, and further to 13.9 Ω for SCS-6. The diffusive resistances (*σ*), which reflect the immediate access of electrolyte ions, are 31.9, 10.3, and 3.3 Ω s^–0.5^ for SCS-2, SCS-4, and SCS-6, respectively (Fig. 4b). Correspondingly, the ion diffusion coefficients (*D*_Zn_^2+^) are 1.5 × 10^−7^ cm^2^ s^−1^ for SCS-2, 1.3 × 10^−7^ cm^2^ s^−1^ for SCS-4, and 1.1 × 10^−6^ cm^2^ s^−1^ for SCS-6 (Table S3). The corresponding time constants (*τ*), representing rapid frequency response, are 55.6, 33.3, and 18.2 s for SCS-2, SCS-4, and SCS-6, respectively (Fig. 4c). The plot of real capacitance (*C*′) versus frequency related to time constant is also keep the same trend with *τ* (Fig. S21a), which is 0.048, 0.10, and 0.20 F for SCS-2, SCS-4, and SCS-6, respectively. The relaxation time constants (*τ*_0_), indicating the temporal duration of energy storage, are 35.7 s for SCS-2, 26.1 s for SCS-4, and 10 s for SCS-6 (Fig. S21b, Table S3). Overall, SCS-6 cathode demonstrates the lowest *R*_s_, *R*_ct_, *σ*, τ_0_ and *τ*, along with the highest $D_{\mathrm{Zn}^{2+}}$, revealing superior surface accessibility and efficient charge transport with the minimal diffusion resistance and exceptional power delivery performance.

The capacity contribution of SCS-*x* cathode from capacitive and diffusion-controlled process was calculated. The power exponent *b*, derived from the equation *i* = *kv*^b^, serves as a crucial indicator of electrochemical kinetics [69, 70], with values closer to 1 suggesting a capacitive-controlled process [71]. The b values are for the cathodic and anodic peaks are 0.81/0.81 for SCS-2 (Fig. 4d) and 0.88/0.86 for SCS-4 (Fig. 4e). In contrast, the *b* values for the cathodic and anodic peaks are 0.96 and 0.95 for SCS-6 (Fig. 4f), indicating a predominantly capacitive-controlled process. In addition, capacitive and diffusion-controlled contributions were further quantified using Dunn’s equation (*i* = *k*_1_ + *k*_2_*v*^0.5^) [72]. At different scan rates (2, 5, 10, 20, 50, 100, and 200 mV s^−1^), SCS-6 cathode consistently exhibits the highest capacitive contributions of 54–95% (Fig. 4g), together with sight diffusion-controlled contribution (Fig. 4h). In summary, the kinetics behavior and capacity contribution analysis reveal that SCS-6 cathode operates via a capacitive-controlled charge storage process, demonstrating efficient electrolyte-ion transport kinetics. The nanosheet subunits of SCS-6 provide abundant adsorption sites and minimized ion transfer distances, while the interconnected mesopores and surface-accessible channels facilitate rapid electrolyte access and effective ion transport.

The intrinsic reaction energy and optical energy gaps of SCS-*x* materials have been evaluated, as critical indicators of the charge storage kinetics in carbon materials. The physical adsorption activation energy (*E*_a1_) is 16.3, 13, and 9.5 kJ mol^−1^ for SCS-2, SCS-4, and SCS-6, respectively (Fig. S22a). Similarly, the redox response activation energy (*E*_a2_), associated with the interaction between heteroatomic sites and electrolyte ions, is found to be 37.6, 32.8, and 19.1 kJ mol^−1^ for SCS-2, SCS-4, and SCS-6, respectively (Fig. S22b). SCS-6 exhibits the lowest physical adsorption (*E*_a1_) and redox response (*E*_a2_) activation energies, indicating superior charge transfer capabilities. Furthermore, SCS-6 displays the narrowest optical energy gap (*E*_g_) of 0.9 eV, compared to 1.8 eV for SCS-2 and 1.4 eV for SCS-4 (Fig. S22c), further indicating its effective charge transfer capabilities. In summary, the combination of the lowest intrinsic reaction energy and the smallest optical energy gaps of SCS-6 underscore its fast charge storage kinetics, making it an optimal material for high-performance energy storage applications.

### Charge Storage Mechanism of Spherical Carbon Superstructures

**Fig. 5:**
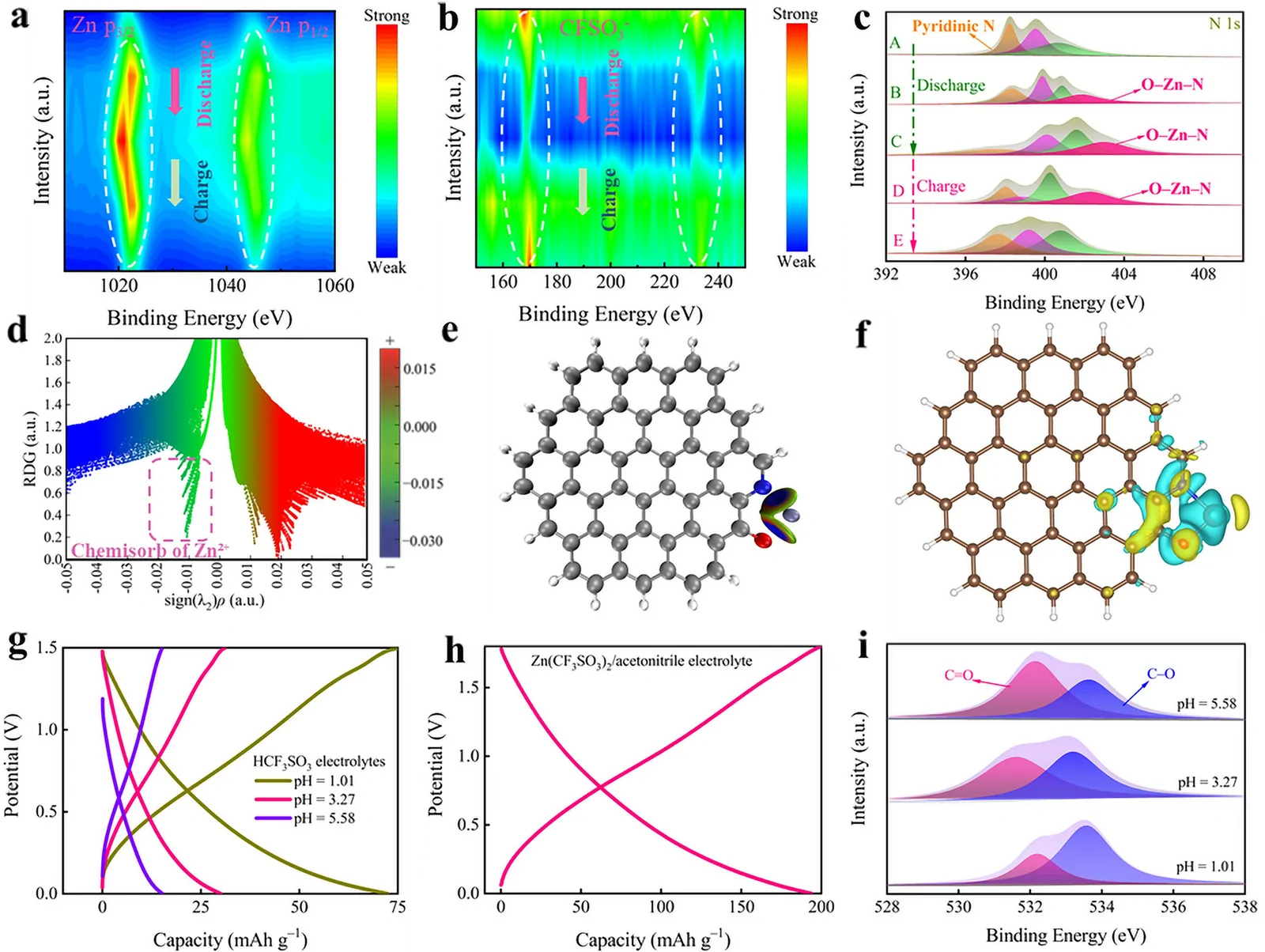
Charge storage mechanism of spherical carbon superstructures. Ex situ XPS spectra at different discharged/charged states of SCS-6 cathode: **a** Zn, **b** S, **c** N. **d** Plots of RDG *vs*. sign(*λ*_2_)*ρ*. **e** Corresponding gradient isosurfaces for both carbonyl O and pyridine N. **f** Differential electron density isosurface of single Zn^2+^ adsorbed on synergetic carbonylic/pyridine sites. **g** GCD curves at 0.2 A g^−1^ of SCS-6 cathode in HCF_3_SO_3_ electrolytes with different pH values. **h** GCD curves at 0.2 A g^−1^ of SCS-6 cathode in 0.5 M Zn(CF_3_SO_3_)_2_/acetonitrile solution. **i** XPS spectra of C 1*s*

To investigate the charge storage mechanism of SCS-6 cathode, ex situ characterizations and density functional theory (DFT) calculations were conducted. SCS-6 cathode was analyzed at various discharge/charge states (A–E), corresponding to specific points on the GCD curve (Fig. S23a). XPS spectra reveal that the intensity of the zinc peaks increases during the discharge process and decreases during the charge process (Figs. 5a and S23b). A similar trend was observed in the XRD patterns, where the intensity of zinc peaks (~ 26.4°) increased during the discharge and decreased during charge (Fig. S23c). Notably, these peaks shift to larger angles during discharge and returns to smaller angles during charge, indicating a reversible precipitation/dissolution process of Zn(CF_3_SO_3_)[Zn(OH)_2_]_3_···*x*H_2_O due to coordination among OH^−^, Zn(CF_3_SO_3_)_2_ and H_2_O (Fig. S23d). These observations suggest that zinc ions adsorb onto SCS-6 cathode during discharge and desorb during charge, confirming a reversible process. In contrast, the intensity of the S element peaks, corresponding to CF_3_SO_3_^−^ ion, exhibits an opposite trend during the discharge/charge process, as demonstrated by XPS spectra (Fig. 5b). This indicates that CF_3_SO_3_^−^ ions adsorb onto SCS-6 cathode during charging and desorb during discharging. Together, these *ex* situ characterizations provide compelling evidence for the reversible adsorption/desorption of Zn^2+^ and CF_3_SO_3_^−^ ions on SCS-6 cathode, underlying the efficient charge storage mechanism of this material.

Chemical redox reactions between Zn^2^⁺ ions and carbonyl/pyridine group, resulting in the formation of O–Zn–N bonds, were identified through an analysis of surface elemental states on the cathode. In N 1*s* spectra (Fig. 5c), curve fitting reveals new peaks at 402.5 eV corresponding to O–Zn–N bonds, indicating that Zn^2^⁺ ions can simultaneously interact with O and N groups. The O 1*s* and C 1*s* spectra further confirm the formation of O–Zn–N bonds. Specifically, the carbonyl group (C=O) peak in O 1*s* spectra increases during charging and decreases during discharging (Fig. S24a), highlighting the electrochemical redox activity of C=O groups and their strong coupling with electrolyte ions. Additionally, C–O–Zn bonds observed in C 1*s* spectra suggest that Zn^2^⁺ ions interact with oxygen groups during discharging (Fig. S24b). DFT calculations support the charge storage mechanism of SCS-6 cathode. RDG plots reveal that Zn^2^⁺ ions chemisorb at carbonyl and pyridine groups (Fig. 5d), as indicated by the branches within the red circle. These interaction areas are located at − 0.02 ~ 0.02 a.u. of sign (*λ*_2_)*ρ*, demonstrating robust coupling between zinc ions and carbonyl/pyridine groups, forming metal–ligand configurations (Figs. 5e and S25). The bonding nature between heteroatomic sites and Zn^2^⁺ ions is further analyzed through differential charge density isosurface simulations (Fig. 5f). The charge depletion around zinc (blue area) and the charge accumulation around carbonyl/pyridine motifs (yellow area) illustrate the charge transfer from zinc ions to these groups. These ex situ characterizations, combined with DFT calculations, reveal a dual-ion uptake mechanism within SCS-6 cathode. This mechanism involves both the chemical and physical adsorption/desorption of Zn^2^⁺ ions as well as the physical desorption of CF_3_SO_3_^−^ ions, highlighting the complex and efficient charge storage processes of this material.

As water decomposes and OH^−^ is consumed, leading to the formation of Zn(CF_3_SO_3_)[Zn(OH)_2_]_3_·*x*H_2_O, it is crucial to investigate the role of H^+^ in Zn^2+^ storage within SCS-6 cathode. Comparison tests are carried out in aqueous HCF_3_SO_3_ electrolyte at different pH values (1.01, 3.27, and 5.58). Notably, since the pH of 3 M Zn(CF_3_SO_3_)_2_ is also 3.27, comparing HCF_3_SO_3_ electrolytes at varying pH levels allows us to isolate and examine the specific effects of H^+^ participation in the Zn^2+^ storage process. These results, showing capacities of 74, 26, and 13 mAh g^−1^ at 0.2 A g^−1^ (Fig. 5g) for pH values of 1.01, 3.27, and 5.58 respectively, strongly indicate that H^+^ plays a significant role in the cathodic electrochemical processes (Fig. S26). Besides, when using a 0.5 M Zn(CF_3_SO_3_)_2_-acetonitrile solution as the electrolyte, in which H^+^ participation is excluded in the electrochemical process, SCS-6 cathode shows a lower capacity of 190 mAh g^−1^ at 0.2 A g^−1^ compared to the capacity observed with Zn(CF_3_SO_3_)_2_ aqueous solution (Fig. 5h). Moreover, H^+^ adsorption facilitates the conversion of activated C=O groups into C–O groups, which subsequently form C–O–H (Fig. 5i). This sequence of reactions underscores the crucial role of H^+^ in energy storage within SCS-6 cathodes through chemical desorption.

Theoretical calculations and ex situ characterization results reveal a proton-assisted dual-ion charge storage mechanism in SCS-6 cathode used in ZHCs. During discharge, Zn^2+^ and H^+^ are stored onto the carbon surface through both physical adsorption and chemical redox interactions. Carbonyl and pyridine groups synergistically facilitate the chemical adsorption of Zn^2+^ by forming O–Zn–N bonds, which enhances the capacity for zinc ion storage. H^+^ reacts with C= O to form C–O–H, thereby contributing additional capacity. During the charge process, CF_3_SO_3_^−^physically adsorb onto the carbon surface, while Zn^2+^ and H^+^ desorb. The charge storage mechanism is summarized as follows:

Cathodes’ chemical redox:

$$ {\mathrm{C}} = {\text{N }} + {\text{ C}} = {\text{O }} + {\text{ Zn}}^{{{2} + }} + {\text{ 2e}}^{ - } \to {\text{ C}} - {\mathrm{O}} - {\mathrm{Zn}} - {\mathrm{N}} - {\mathrm{C}} $$

$$ {\mathrm{C}} = {\mathrm{O}} + {\mathrm{H}}^{ + } + {\mathrm{e}}^{ - } \to {\mathrm{C}} - {\mathrm{O}} - {\mathrm{H}} $$

Cathodes’ physical adsorption/desorption:

$$ {\mathrm{C}} + {\mathrm{Zn}}^{{{2} + }} \to {\text{ Zn}}^{{{2} + }} ||{\mathrm{C}} + {\mathrm{H}}^{ + } \to {\mathrm{C}}||{\mathrm{H}}^{ + } $$

$$ {\mathrm{Anodes}}\;{\mathrm{chemical}}\;{\mathrm{redox:}}\;\;{\mathrm{Zn}} \to {\mathrm{Zn}}^{{{2} + }} + {\mathrm{2e}}^{ - } $$

## Conclusion

A hydrogen-bond-oriented interfacial super-assembly route is developed to customize SCSs for activating superior Zn-ion storage with double-high capacitive activity and durability. Tetrachlorobenzoquinone, as a H-bond acceptor, interacts with dimethylbenzidine, as a H-bond donator, to form organic nanosheet modules, which are sequentially assembled, orientally compacted and densified into well-orchestrated superstructures through multiple H-bonds (N–H···O). Featured with surface-active heterodiatomic motifs, open nanoporous channels, and successive charge migration pathways, SCSs cathode promises the high accessibility of built-in zincophilic sites and rapid ion diffusion with low energy barriers. As a result, the assembled Zn||SCSs capacitor harvests all-round improvement in Zn-ion storage metrics, including high energy density, high-rate performance and long-lasting cycling lifespan. An opposite charge-carrier storage mechanism is rationalized for SCSs cathode to maximize spatial capacitive charge storage, involving high-kinetics physical Zn^2+^/CF_3_SO_3_^−^ adsorption and chemical Zn^2+^ redox with carbonyl/pyridine groups. This work provides valuable insights into hydrogen-bond-driven interfacial super-assembly strategies for designing superstructural carbons, paving the way for advanced energy storage technologies.

## Supplementary Information

Below is the link to the electronic supplementary material.

Supplementary file1 (DOCX 8833 KB)
