# Supplementary material for: Hydrogen-Bonded Interfacial Super-Assembly of Spherical Carbon Superstructures for High-Performance Zinc Hybrid Capacitors
Source: Nanomicro Lett. 2025 Aug 25;18:38. doi: 10.1007/s40820-025-01883-1 (PMC12378267; doi:10.1007/s40820-025-01883-1)
Supplement: Supplementary file 1 — Supplementary file1 (DOCX 8833 KB) [file 40820_2025_1883_MOESM1_ESM.docx]

Supporting Information for

**Hydrogen-Bond****ed Interfacial** **Super-Assembly of Spherical Carbon Superstructures for** **High-Performance** **Zinc Hybrid Capacitors**

Yang Qin^1^, Chengmin Hu^3^, Qi Huang^4^, Yaokang Lv^5^, Ziyang Song^1, 2,^ *, Lihua Gan^1, 6,^ *, Minxian Liu^1, 6,^ *

^1^ Shanghai Key Lab of Chemical Assessment and Sustainability, School of Chemical Science and Engineering, Tongji University, 1239 Siping Rd., Shanghai 200092, P. R. China

^2^ State Key Laboratory of Pollution Control and Resource Reuse, College of Environmental Science and Engineering, Advanced Research Institute, Tongji University, 1239 Siping Rd., Shanghai, 200092, P. R. China

^3^ Department of Chemistry, Shanghai Key Lab of Molecular Catalysis and Innovative Materials and Collaborative Innovation Center of Chemistry for Energy Materials, Fudan University, 2005 Songhu Rd., Shanghai 200438, P. R. China

^4^ Institute for Electric Light Sources, School of Information Science and Technology, Fudan University, 2005 Songhu Rd., Shanghai 200438, P.R. China

^5^ College of Chemical Engineering, Zhejiang University of Technology, 18 Chaowang Rd., Hangzhou 310014, P. R. China

^6^ State Key Laboratory of Cardiovascular Diseases and Medical Innovation Center, Shanghai East Hospital, School of Medicine, Tongji University, 150 Jimo Rd., Shanghai, 200120, P. R. China

*Corresponding authors. E-mail: [songziyang@tongji.edu.cn](mailto:songziyang@tongji.edu.cn) (Ziyang Song); [ganlh@tongji.edu.cn](mailto:ganlh@tongji.edu.cn) (Lihua Gan), [liumx@tongji.edu.cn](mailto:liumx@tongji.edu.cn) (Minxian Liu)

**Supplementary Figures and Tables**

**
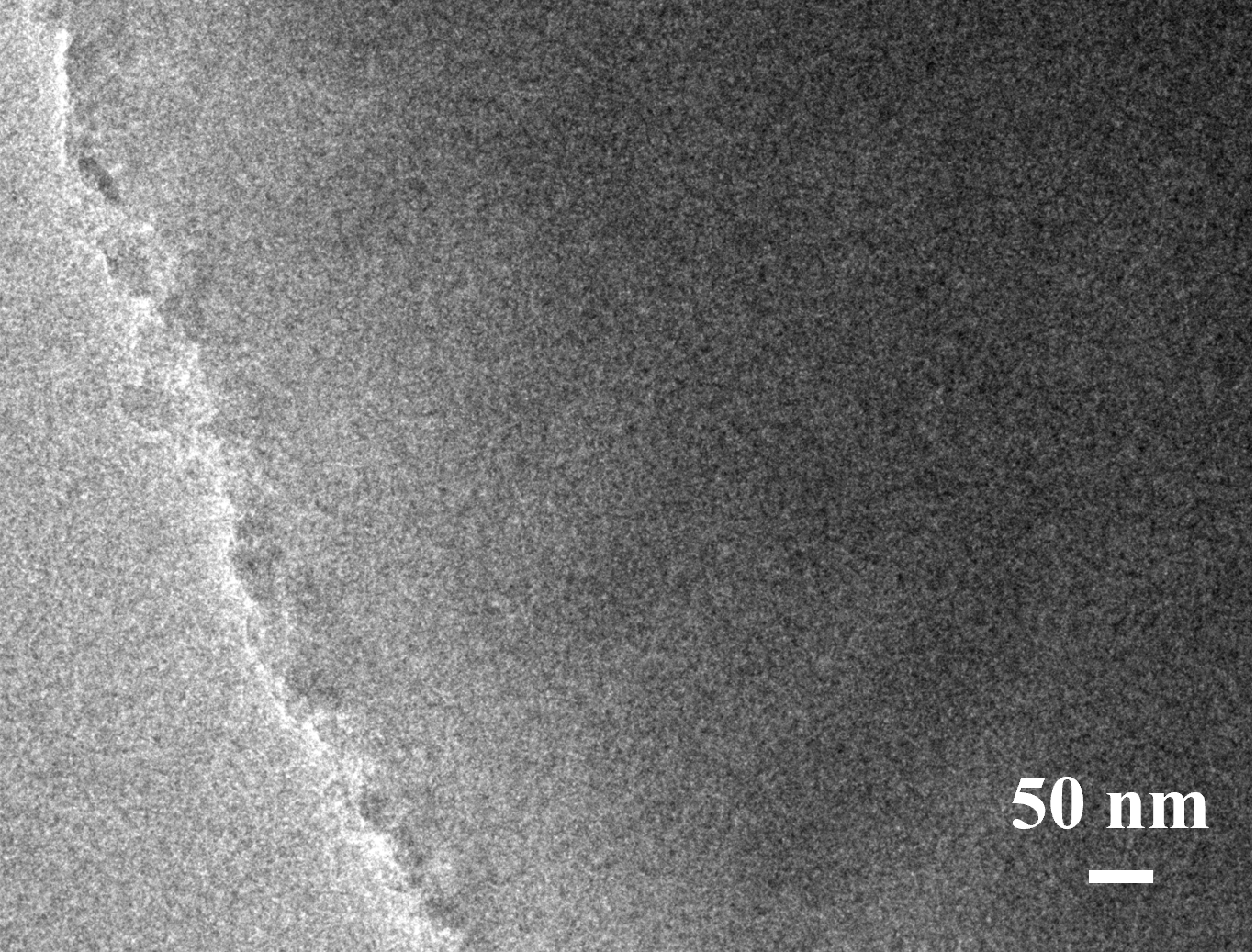
**

**Fig. S1** TEM image of SCS-6

**
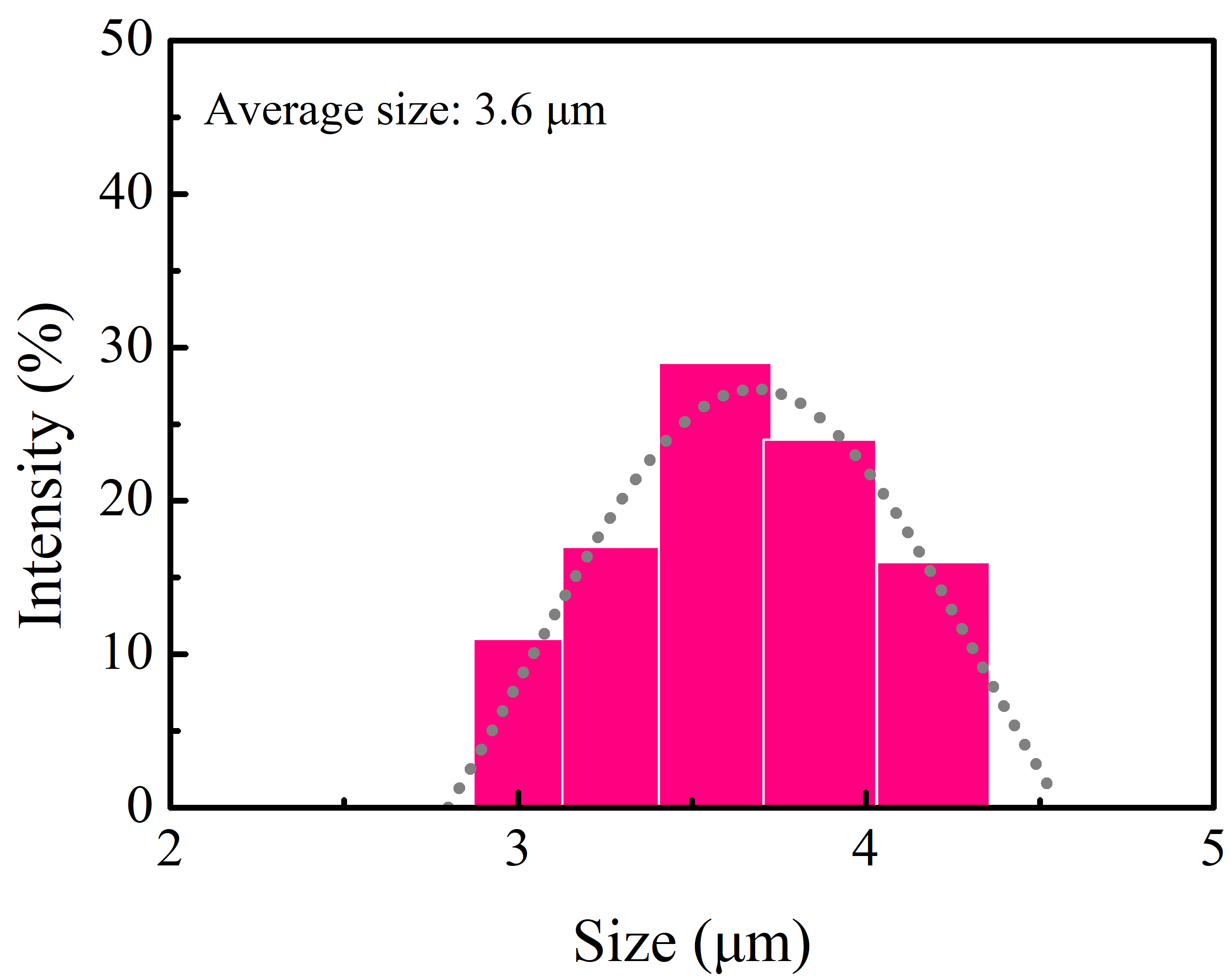
**

**Fig. S2** Particle size distribution of SCS-6

**
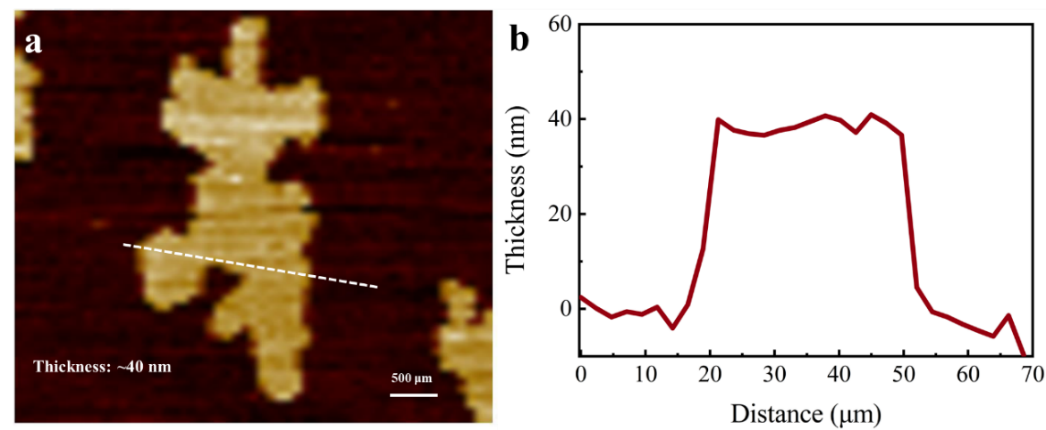
**

**Fig. S3 a−b** Atomic force microscopy test

**
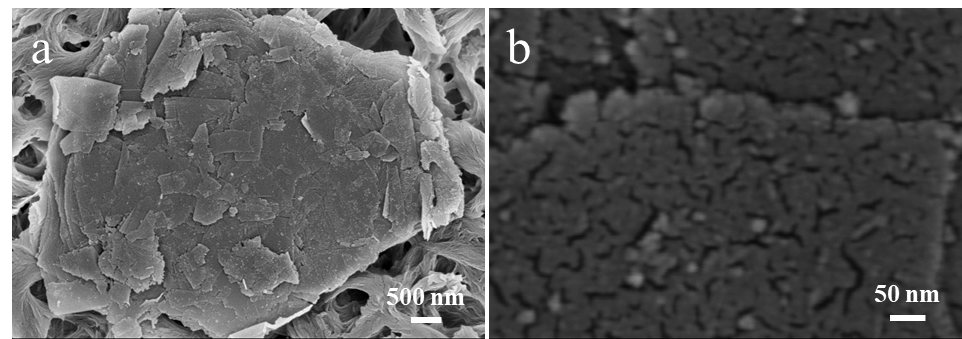
**

**Fig. S4 a−b** SEM images of SPS-0.5

**
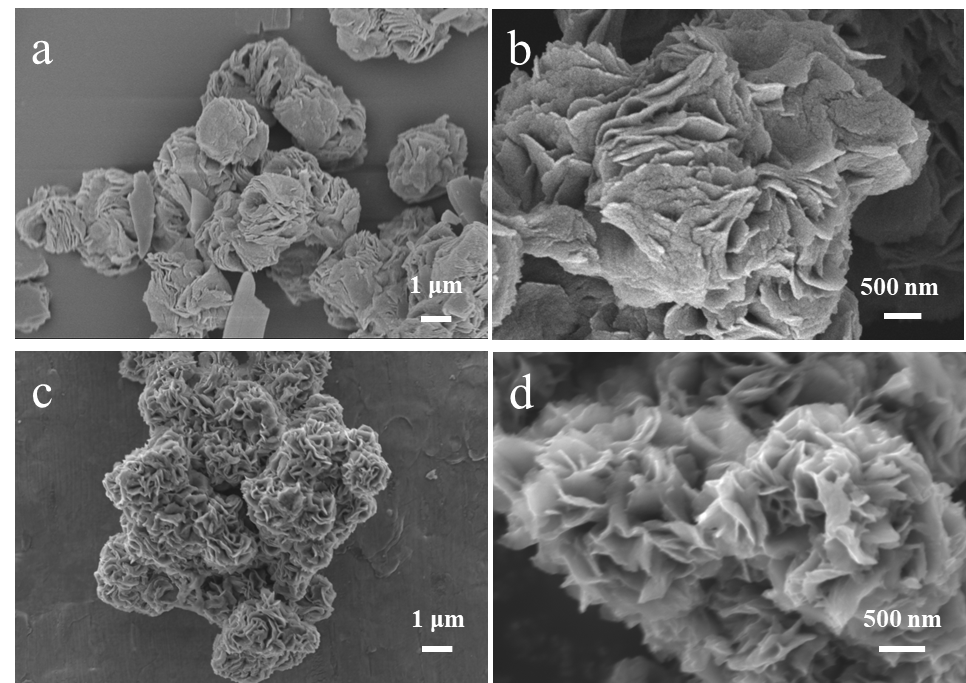
**

**Fig. S5 a−b** SEM images of SPS synthesized in DMF and **c−d** DMAc solvent

**
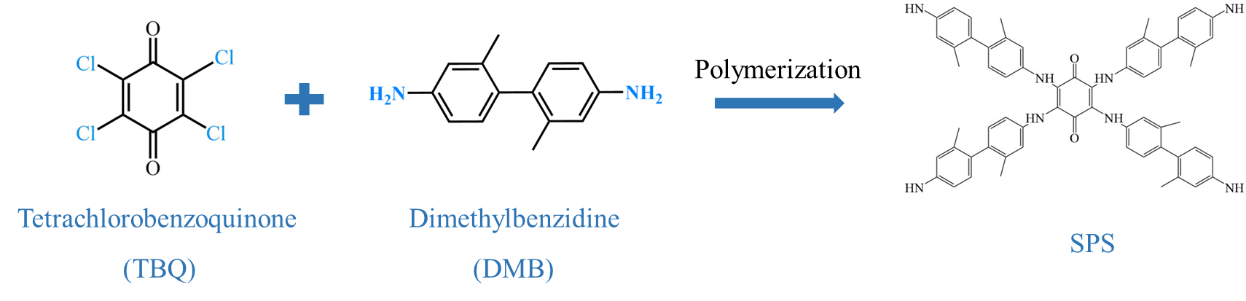
**

**Fig. S6** Synthesis route of precursors

**
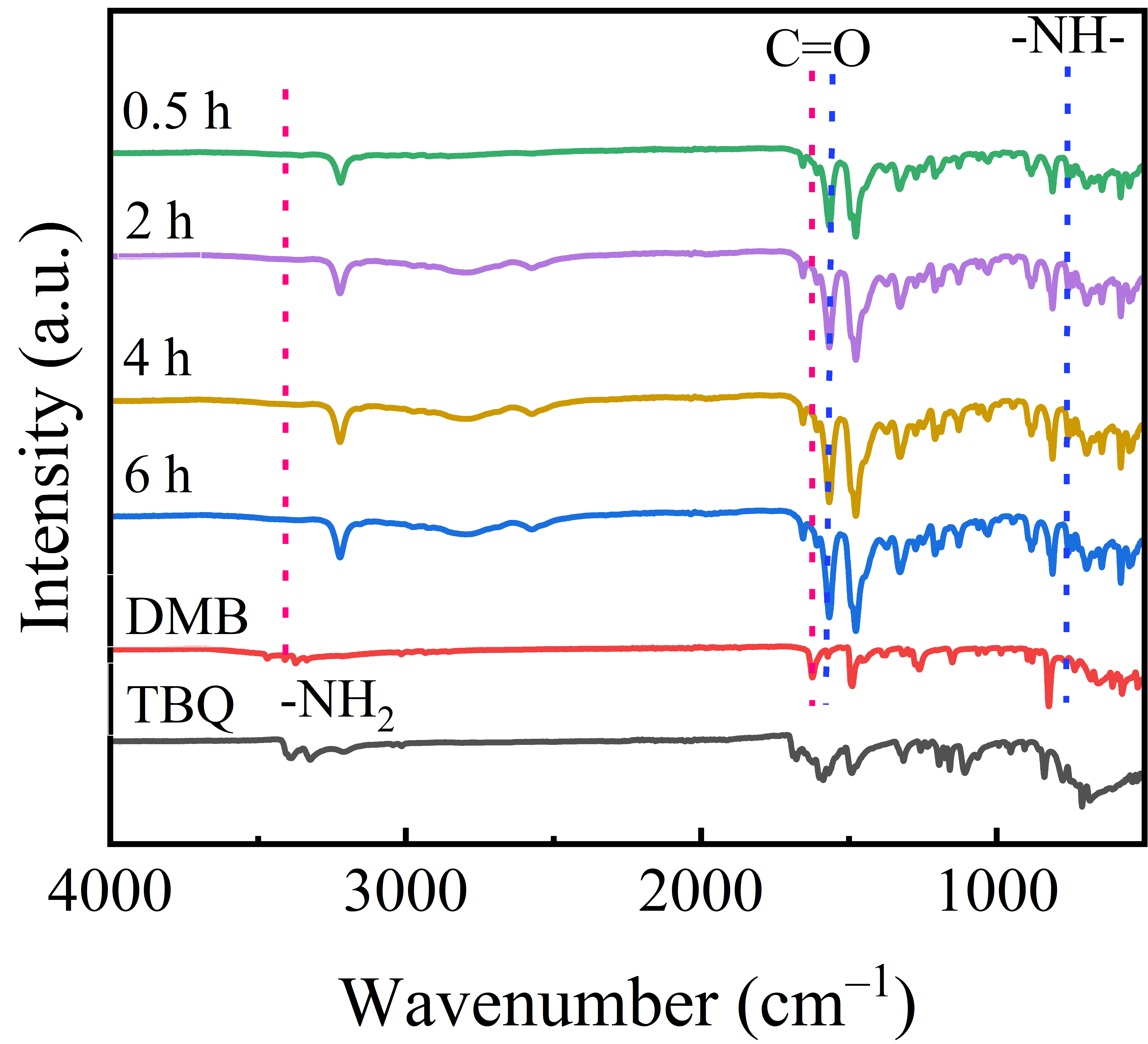
**

**Fig. S7** FT-IR spectra of SPS-*x* and raw materials


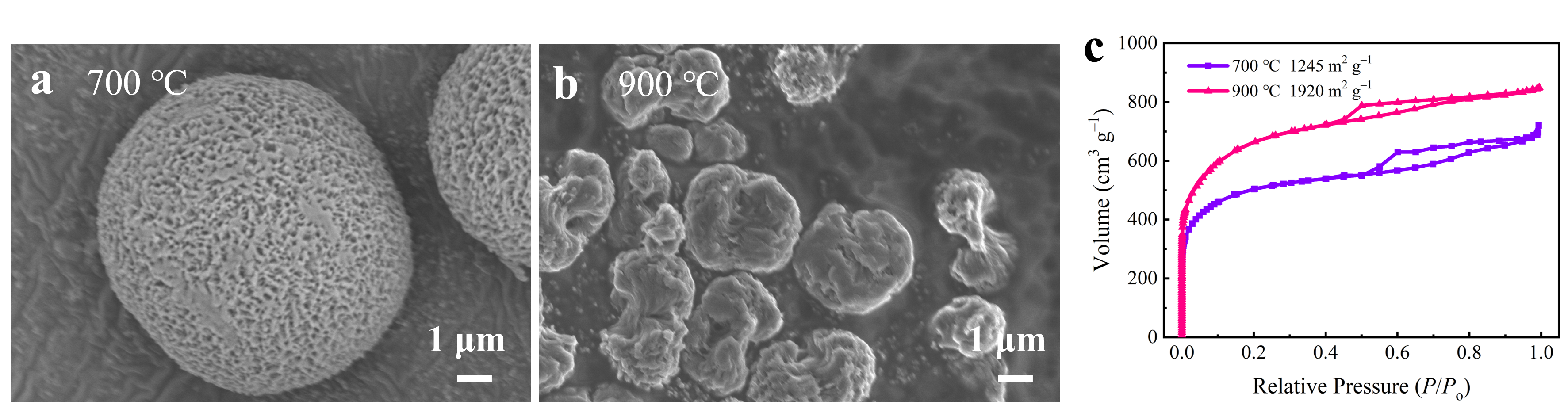


**Fig. S8 a** and **b** SEM images of SCS-6 obtained by pyrolysis at 700 ℃ and 900 ℃. **c** Nitrogen absorption/desorption isotherms.

**Note:** Compared with SCS-6 obtained from pyrolysis at 800 ℃ (Fig. 1b and Fig. 3a), SCS-6 at 700 ℃ shows well-maintained spherical superstructures (Fig. S8a) but with a relatively low surface area of 1245 m^2^ g^−1^ (Fig. S8b) due to insufficient carbonization/activation of H-bonding super-assembled polymers. Nevertheless, a higher pyrolysis temperature (900 °C) brings skeleton deformation and excessively etches the H-bonding polymeric matrix, resulting in collapsed superstructures and decreases the surface area to 1920 m^2^ g^−1^. Thus, the pyrolysis temperature was fixed at 800 °C to custom-tailor well-arranged spherical carbon superstructures with desirable structure and function towards efficient energy storage.

**Table S1** Pore structure parameters of SCS-*x* samples

| **Samples** | **SSA**  **(m^2^ g^-1^)** | ***S*_micro_**  **(m^2^ g^-1^)** | ***S*_meso_**  **(m^2^ g^-1^)** | ***V*_total_**  **(m^2^ g^-1^)** | ***V*_meso_/*V*_total_**  **(%)** |
| --- | --- | --- | --- | --- | --- |
| SCS-2 | 1801 | 1553.8 | 247.6 | 1.1 | 40 |
| SCS-4 | 2092 | 1607.9 | 484.2 | 1.6 | 53 |
| SCS-6 | 2530 | 1482.6 | 1047.2 | 2.2 | 70 |

*S*_micro_, *S*_meso_, *V*_total_ and *V*_meso_ represent surface areas, micropore surface areas, mesopore surface areas, total pore volumes and mesopore volumes, respectively.

**
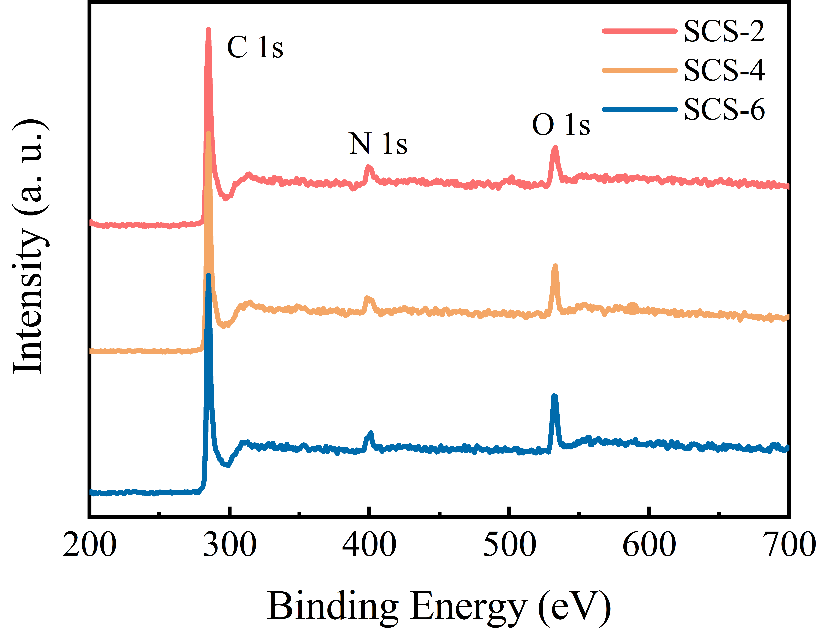
**

**Fig. S9** XPS full spectra of SCS-*x*

**
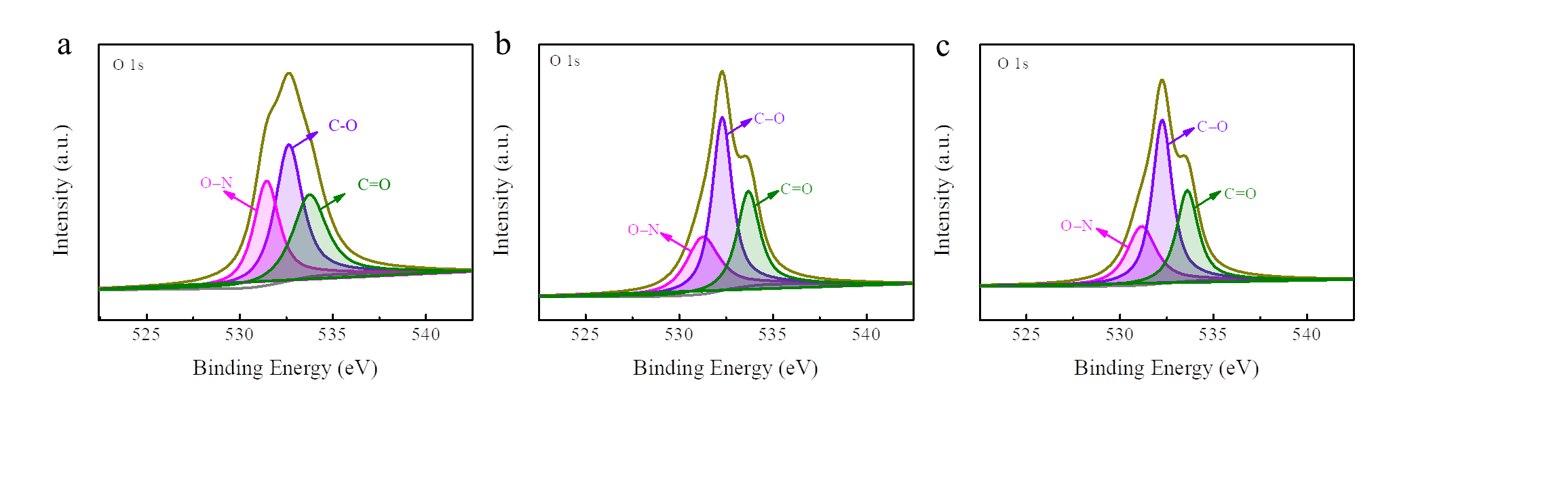
**

**Fig. S10** Fitted high-resolution XPS spectra of O 1s of **a** SCS-2, **b** SCS-4, **c** SCS-6

**
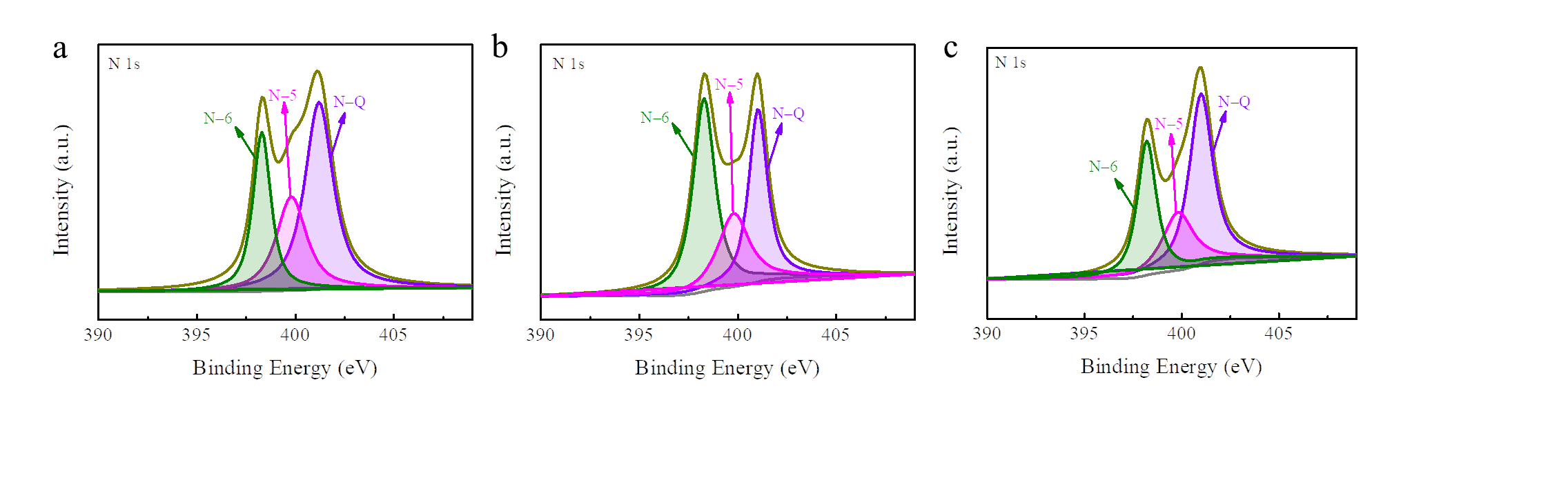
**

**Fig. S11** Fitted high-resolution XPS spectra of N 1s of **a** SCS-2, **b** SCS-4, **c** SCS-6

**
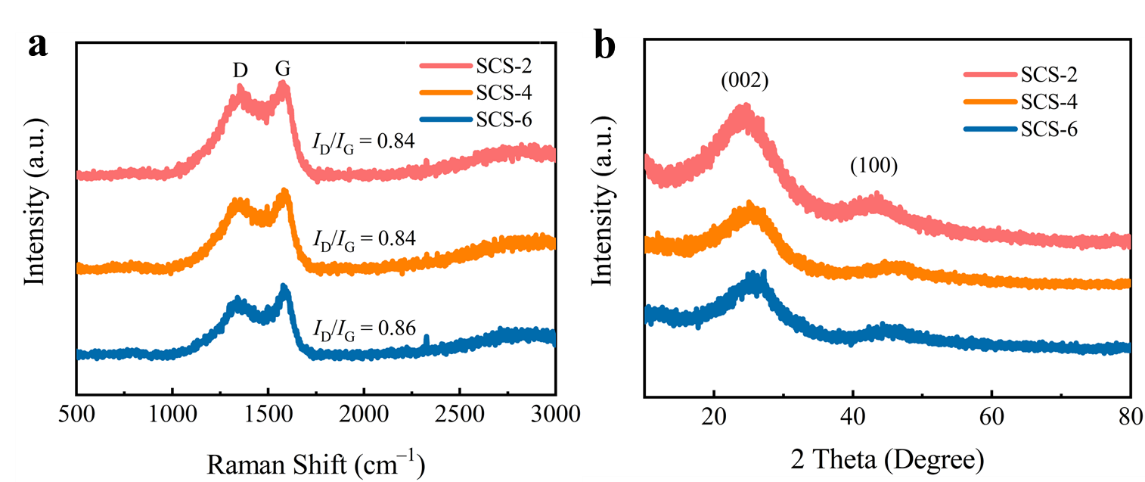
**

**Fig. S12 a** Raman spectra of SCS-*x*. **b** XRD patterns of SCS-*x*


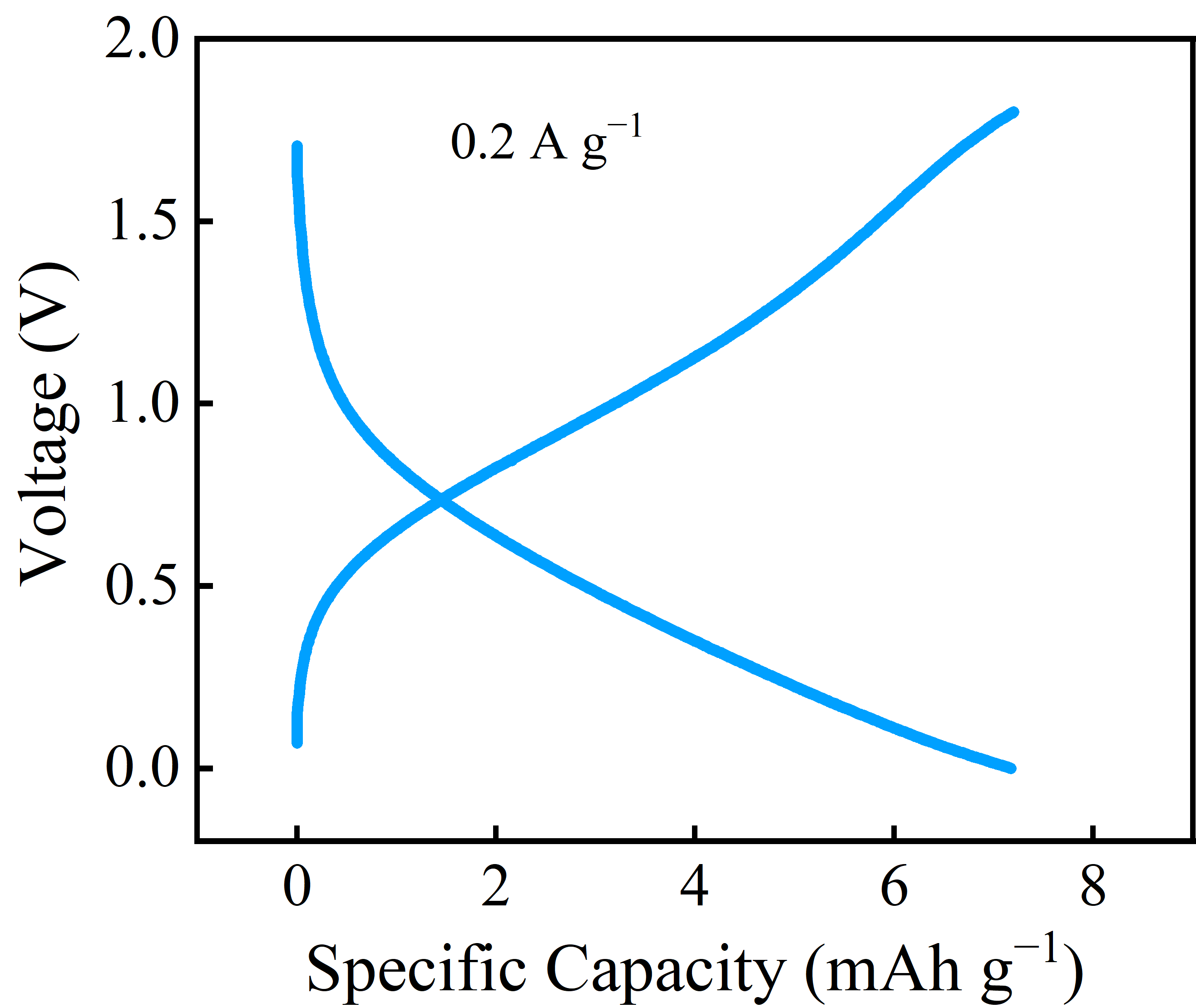


**Fig. S13** The specific capacity of graphite conductive agent


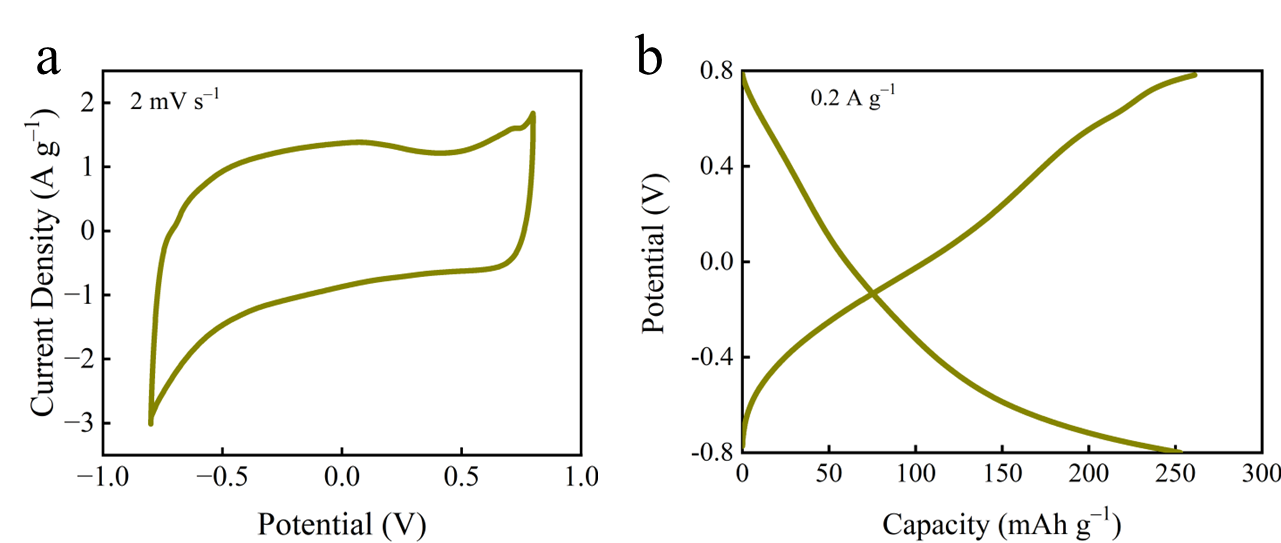
**
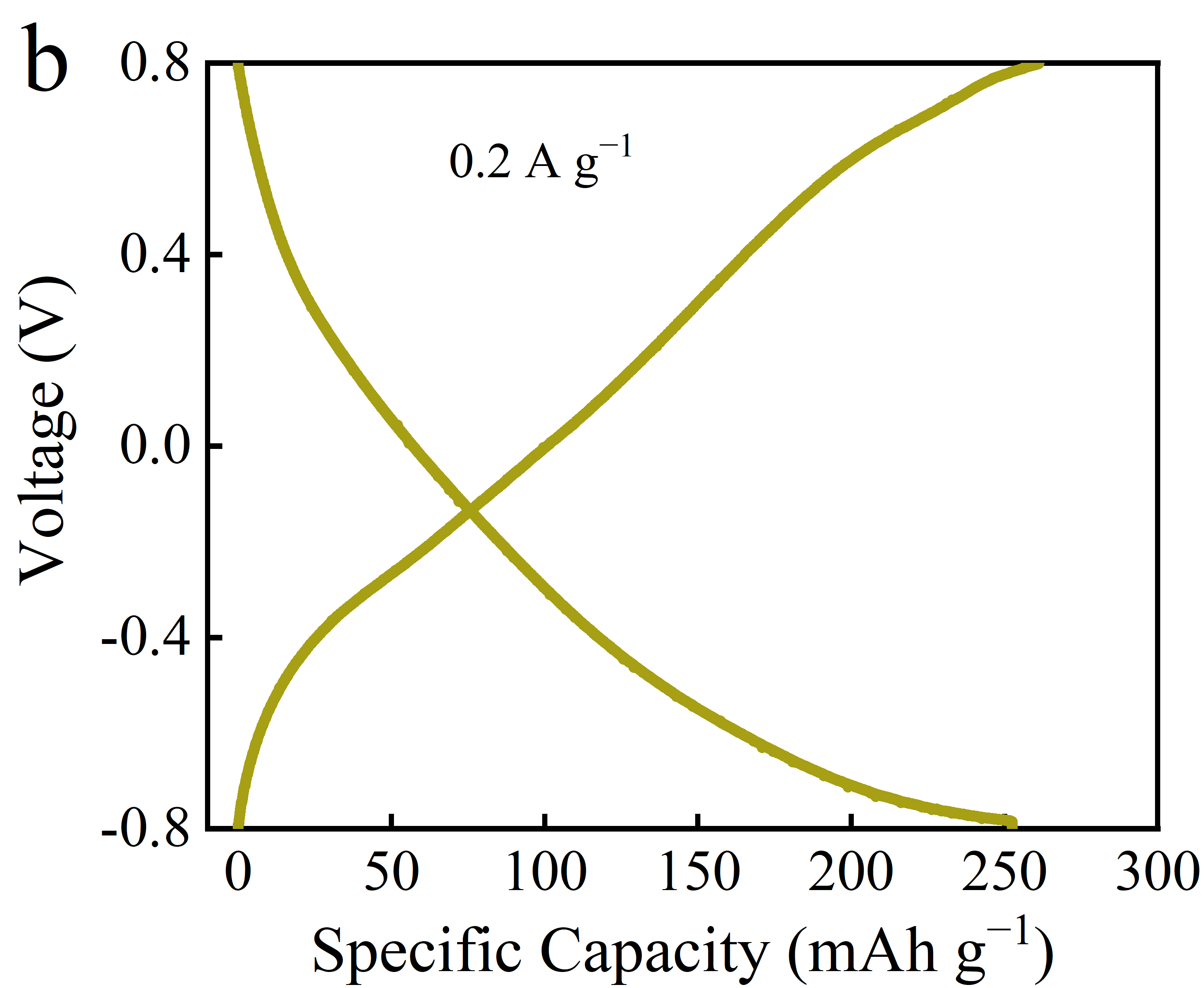
**

**Fig. S14** **a** CV curve and **b** GCD profile of SCS-6 electrode in a three-electrode system using aqueous Zn(CF_3_SO_3_)_2_ electrolyte (Ag/AgCl as the reference electrode, Pt as the counter electrode).

**Note:** CV profile of SCS-6 electrode shows quasi-rectangular shape at 2 mV s^−1^ (Fig. S14a), which indicates the desirable capacitive storage behavior with a capacity of 252 mAh g^−1^ in GCD curve at 0.2 A g^−1^ (Fig. S14b). These results are consistent with the performances of the full-cell configuration (Fig. 3d and e)


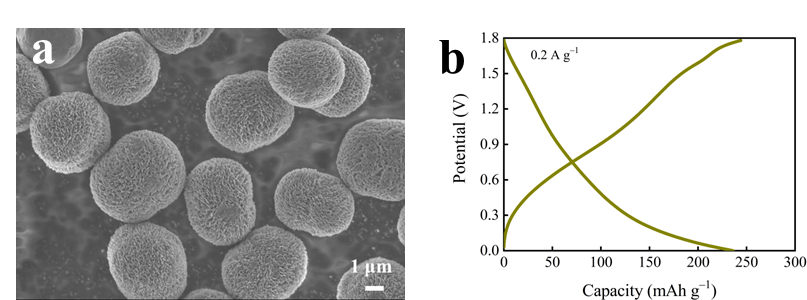


**Fig. S15** **a** SEM image and **b** GCD curve of SCS-8

**Note:** SPS-8 exhibits the same morphology (Fig. S15a) as SPS-6 (Fig. 1b−d), indicating that the monodisperse 3D spherical superstructures of SPS-6 is the final form. SPS-8 cathode delivers a specific capacity of 242 mAh g^−1^ at 0.2 A g^−1^ (Fig. S15b), which is slightly lower than SCS-6 cathode (246 mAh g^−1^, Fig. 3e). These results indicate that SPS-6 obtained at the reaction time of 6 h is the optimal

**
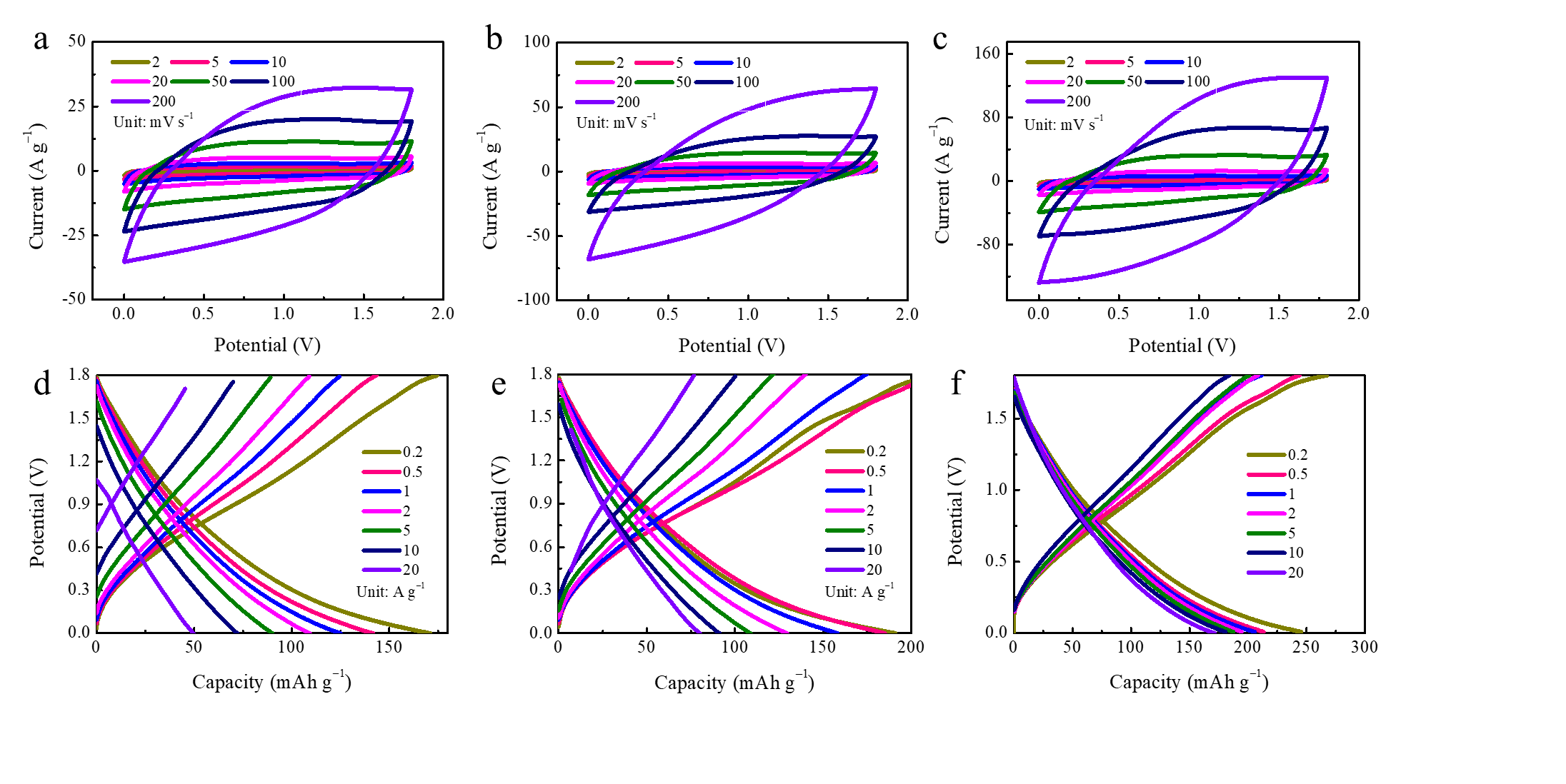
**

**Fig. S16** Electrochemical performances of SCS-*x* electrodes: GCD curves of **a** SCS-2, **b** SCS-4 and **c** SCS-6; CV curves of **d** SCS-2, **e** SCS-4 and **f** SCS-6

**
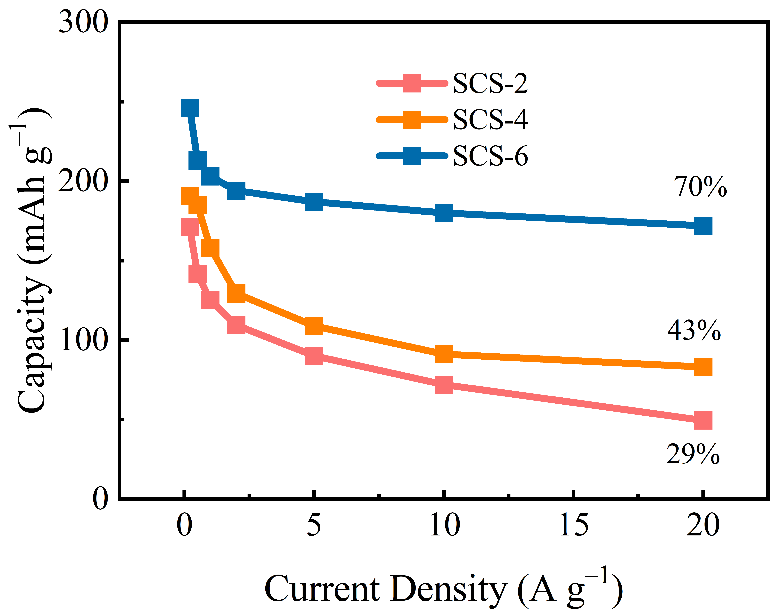
**

**Fig. S17** Rate capabilities of SCS-*x* electrodes ranging from 0.2−20 A g^−1^

**Table S2** Electrochemical performances of ZIHCs

| **Carbon electrodes** | **Electrolyte** | **Voltage**  **(V)** | ***E***  **(Wh kg^-1^)** | **Cycling**  **performance** | **References** |
| --- | --- | --- | --- | --- | --- |
| NP-HPC_3_ | 1 M Zn(CF_3_SO_3_)_2_ | 1.7 | 107.8 | 96.9%  (10, 000 cycles) | [S1] |
| SA-3 | 1 M ZnSO_4_ | 1.6 | 100 | 93%  (500, 00 cycles) | [S2] |
| PSR-4 | 3 M Zn(CF_3_SO_3_)_2_ | 1.8 | 66.16 | 85%  (8, 000 cycles) | [S3] |
| LHPC-700 | 2 M ZnSO_4_ | 1.6 | 63.5 | 88%  (8, 000 cycles) | [S4] |
| HPC-4 | 2 M ZnSO_4_ | 1.6 | 100.56 | 101.86%  (5, 000 cycles) | [S5] |
| CNS-2 | 3 M Zn(CF_3_SO_3_)_2_ | 1.8 | 163 | 93%  (200, 000 cycles) | [S6] |
| HC-Co-F1 | 2 M ZnSO_4_ | 1.6 | 144.2 | 82.4%  (2500 cycles) | [S7] |
| N-RGO/AAQ | 2 M ZnSO_4_ | 1.8 | 122.9 | 93.3%  (12, 000 cycles) | [S8] |
| CMF-800 | 1 M ZnSO_4_ | 1.8 | 87 | 98.3%  (10, 000 cycles) | [S9] |
| NPCNs | 1 M Zn(CF_3_SO_3_)_2_ | 1.8 | 143 | 95.5%  (20, 000 cycles) | [S10] |
| **SCS-6** | **3 M Zn(CF_3_SO_3_)_2_** | **1.8** | **166** | **95.5%**  **(300, 000 cycles)** | **This work** |


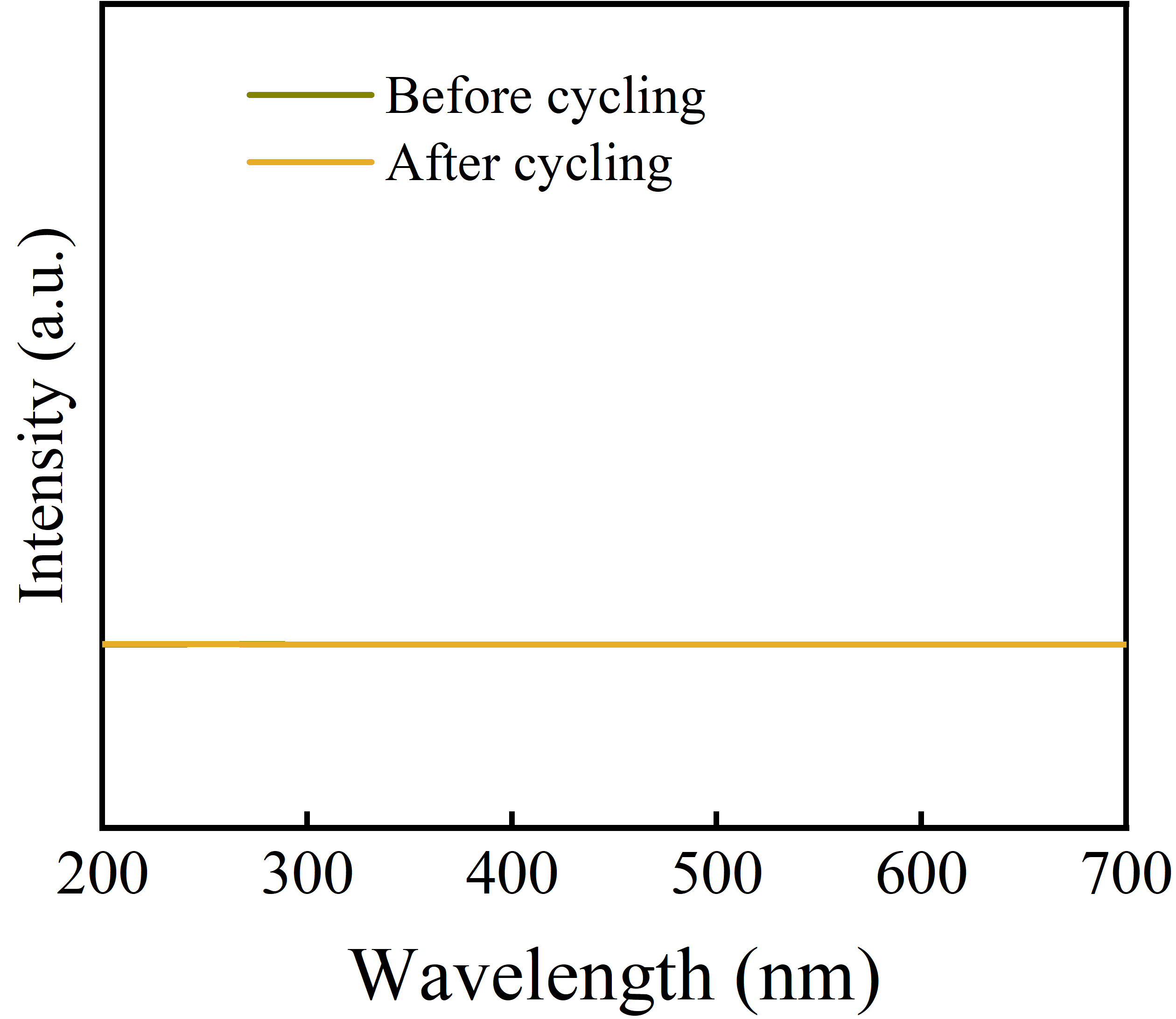


**Fig. S18** UV-vis analysis of aqueous Zn(CF_3_SO_3_)_2_ electrolyte before and after cycling

**Note:** Considering that tetrachlorobenzoquinone with multiple -C=O groups may be water-soluble, we performed UV-vis analysis on aqueous Zn(CF_3_SO_3_)_2_ electrolyte before and after cycling of SCS-6 cathode (Fig. S18). There is no UV-vis adsorption signal of C=O groups from tetrachlorobenzoquinone for aqueous Zn(CF_3_SO_3_)_2_ electrolyte before and after cycling (Fig. S18), suggesting that SCS-6 cathode is very stable in the electrolyte during the electrochemical process.


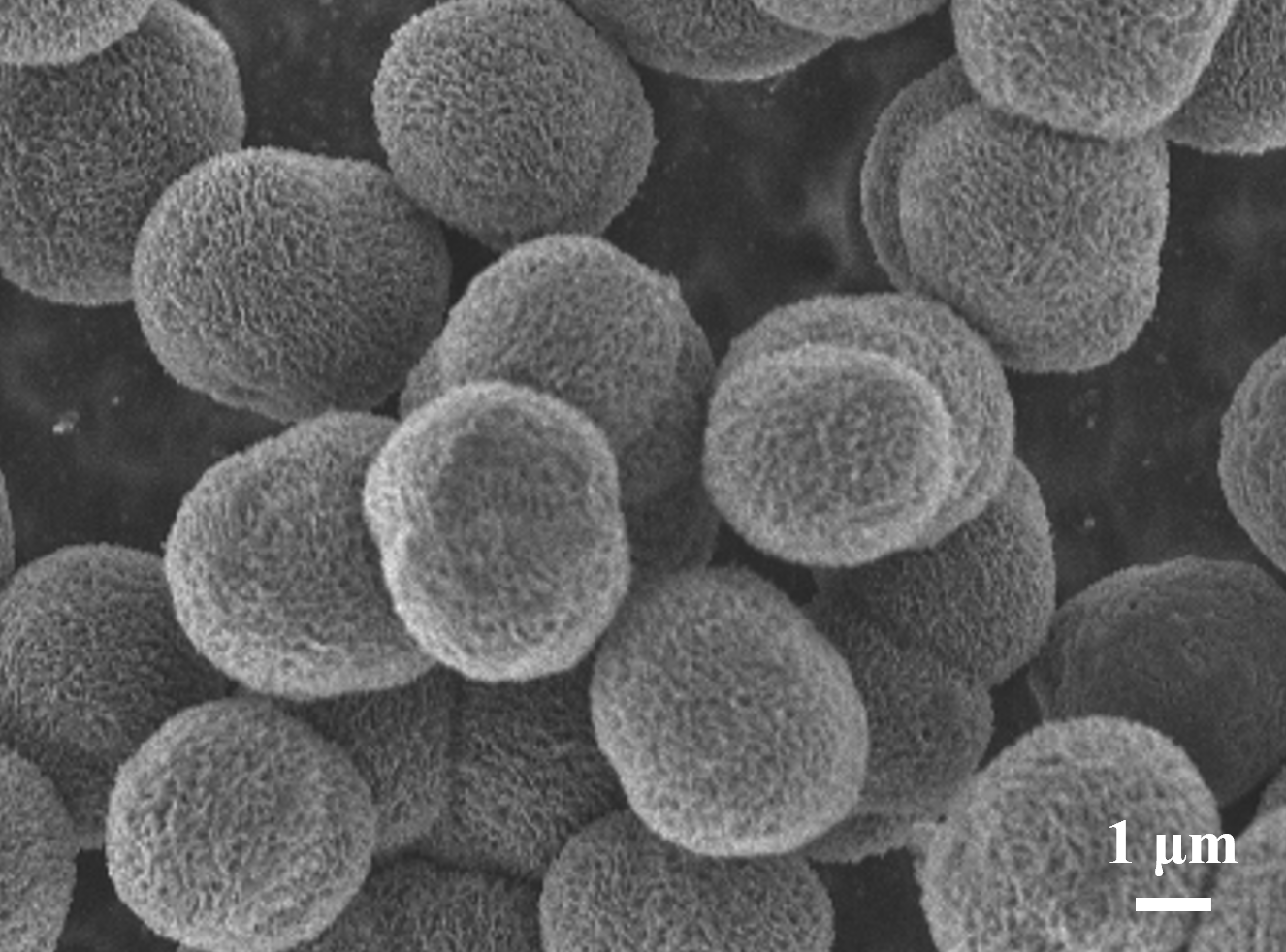


**Fig. S19** SEM image of SCS-6 cathode


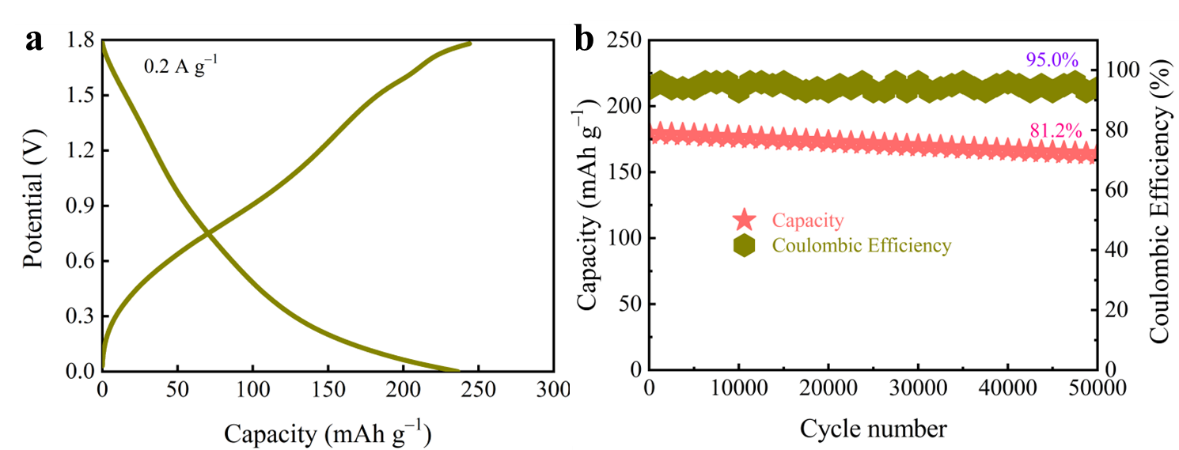


**Fig. S20 a** GCD profile and **b** cycling stability of Zn||SCS-6 capacitors with high-mass-loading SCS-6 cathode (10.2 mg cm^−2^)

**Table S3.** *R*_s_, *R*_ct_, *τ*_0_, *τ, σ* and *D*_Zn_^2+^ of HCS*-x* electrodes

| **Samples** | ***R*_s_ (Ω)** | ***R*_ct_ (Ω)** | ***τ* (s)** | ***σ* (Ω s^–0.5^)** | ***D*_Zn_^2+^ (cm^2^ s^−1^)** | ***τ*_0_ (s)** |
| --- | --- | --- | --- | --- | --- | --- |
| SCS-2 | 7 | 45.4 | 55.6 | 31.9 | 1.5×10^−7^ | 35.7 |
| SCS-4 | 5.7 | 27.8 | 33.3 | 10.3 | 1.3×10^−7^ | 26.1 |
| SCS-6 | 3.5 | 13.9 | 18.2 | 3.3 | 1.1×10^−6^ | 10 |

**
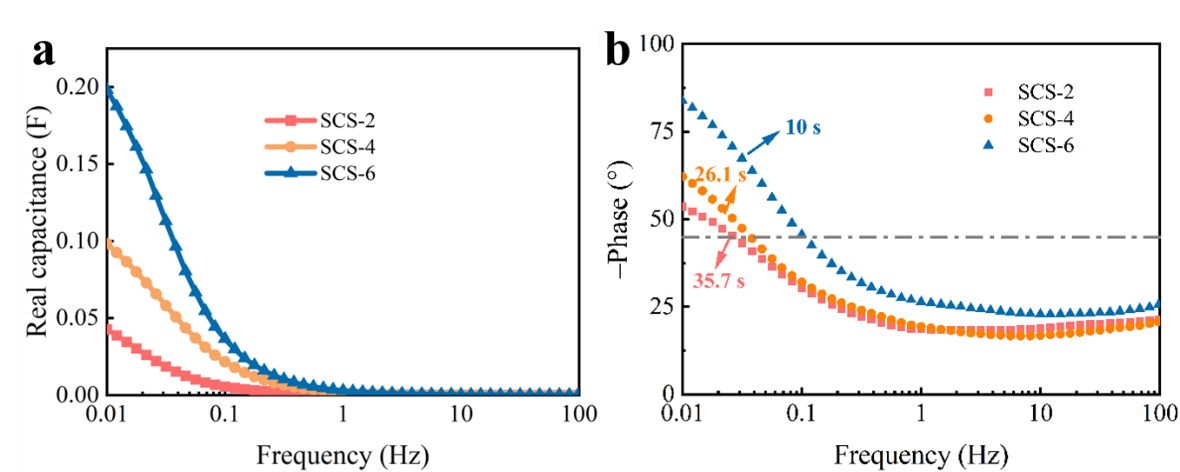
**

**Fig. S21 a** Real capacitance (*C*′) versus frequency. **b** Bode phase diagram

**
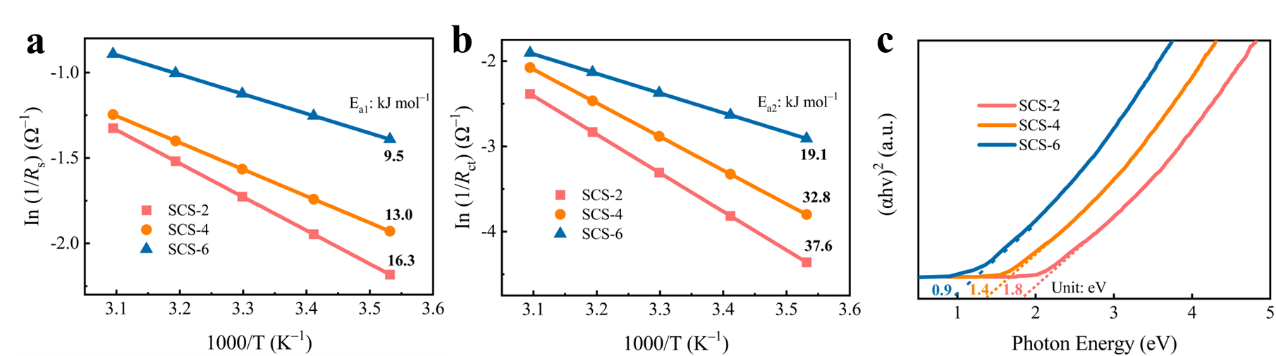
**

**Fig. S22 a** Activation energy of physical adsorption. **b** Activation energy of chemical adsorption. **c** Optical energy gaps

**
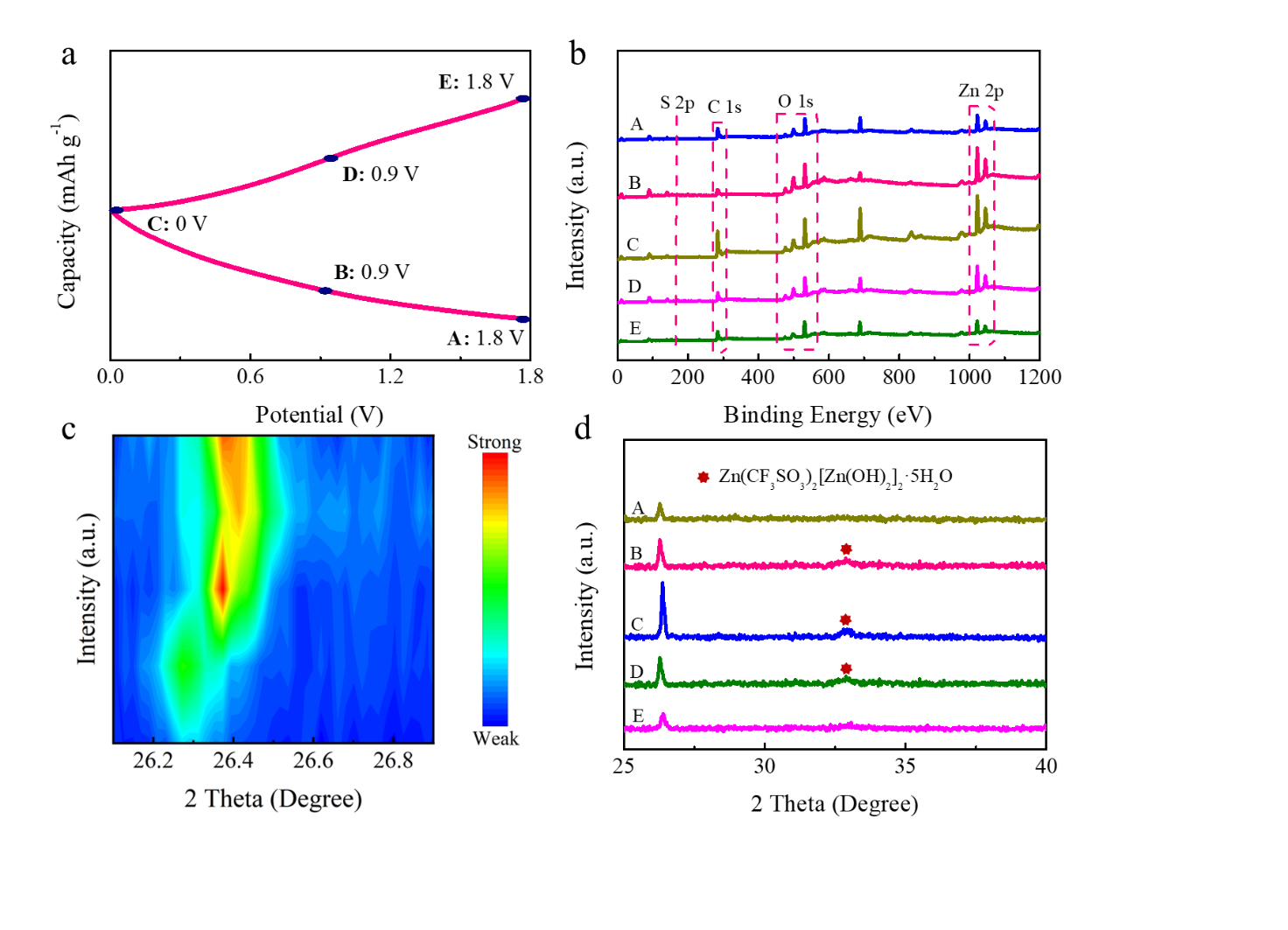
**

**Fig. S23 a** Five representative points corresponding to the GCD profile. **b-c** *Ex*-*situ* XRD patterns. **d** *Ex*-*situ* XPS spectra

**
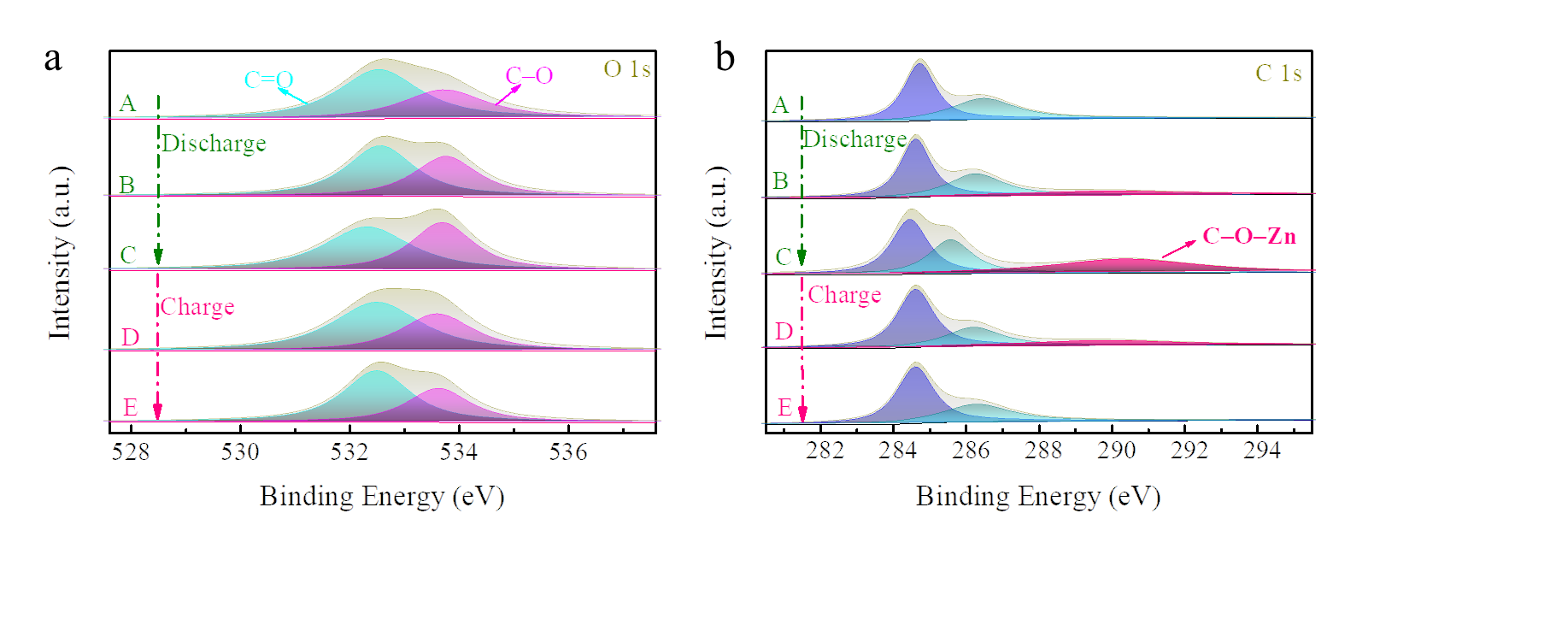
**

**Fig. S24** *Ex-situ* XPS spectra of O 1s and C 1s

**
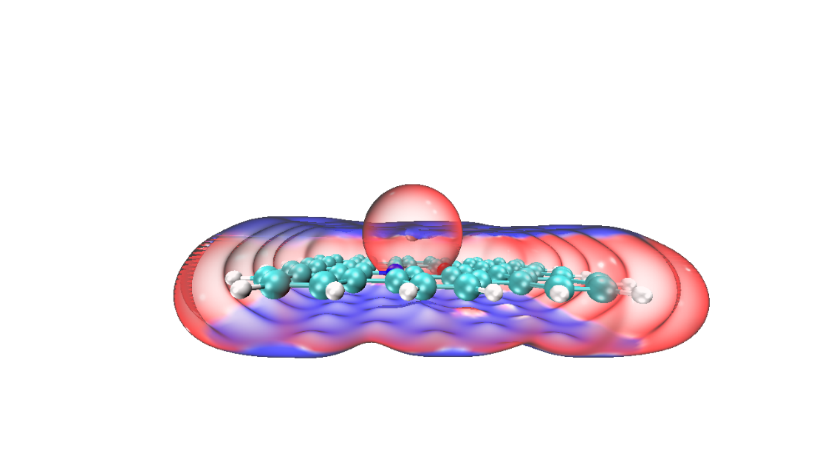
**

**Fig. S25** The model of the interaction between Zn^2+^ and redox motifs


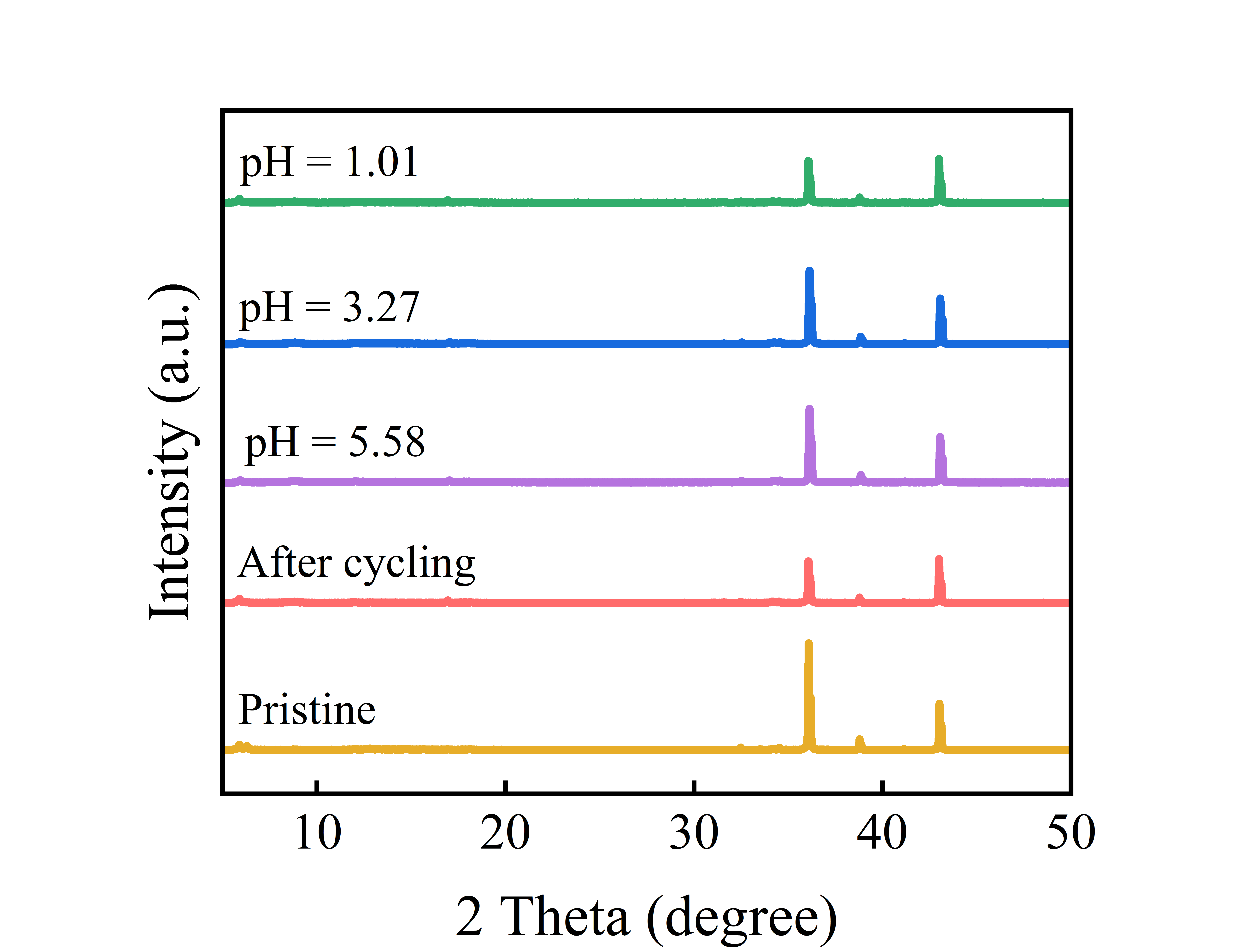


**Fig. S26** XRD patterns of Zn at different conditions

**Notes:** XRD characterization was performed to monitor the structural variation of Zn anodes in HCF_3_SO_3_ electrolytes with different pH values (Fig. S26). Compared with the original Zn foil, no impurity peaks are detected for Zn anodes in various acidic regions (pH=1.01~5.58). Even after consecutive 500,000 (dis)charge cycles, the Zn anode still shows typical signals without obvious dissolution and byproduct formation. These results confirm the robust structural stability of the Zn anode, which allows the high compatibility with SCS-6 cathode for propelling high-performance zinc hybrid capacitors.

**Supplementary References**

1. S. Gao, F. Wei, X. Zhu, Z. Tai, J. Qiao, Electronically modified hierarchical porous carbon by N, P heteroatoms for zinc ion hybrid capacitor. Appl. Surf. Sci. **644**, 158758 (2024). <https://doi.org/10.1016/j.apsusc.2023.158758>
2. X. Pan, Q. Li, T. Wang, T. Shu, Y. Tao, Controllable synthesis of electric double-layer capacitance and pseudocapacitance coupled porous carbon cathode material for zinc-ion hybrid capacitors. Nanoscale **16**(7), 3701–3713 (2024). <https://doi.org/10.1039/D3NR06258A>
3. Z. Sun, X. Jiao, S. Chu, Z. Li, Low-cost porous carbon materials prepared from peanut red peels for novel zinc-ion hybrid capacitors. ChemistrySelect **8**(47), e202304071 (2023). <https://doi.org/10.1002/slct.202304071>
4. J. Ma, S. Yang, T. Huang, X. Zu, Y. Sun et al., 3D hierarchical tri-doped porous carbon derived from calcium lignosulfonate for high-performance zinc ion hybrid capacitors. New J. Chem. **47**(37), 17549–17557 (2023). <https://doi.org/10.1039/D3NJ03537A>
5. F. Mo, Y. Wang, T. Song, X. Wu, Nitrogen and oxygen Co-doped hierarchical porous carbon for zinc-ion hybrid capacitor. J. Energy Storage **72**, 108228 (2023). <https://doi.org/10.1016/j.est.2023.108228>
6. Y. Qin, S. Jha, C. Hu, Z. Song, L. Miao et al., Hydrogen-bonded micelle assembly directed conjugated microporous polymers for nanospherical carbon frameworks towards dual-ion capacitors. J. Colloid Interface Sci. **675**, 1091–1099 (2024). <https://doi.org/10.1016/j.jcis.2024.07.052>
7. L. Huang, D. Tong, H. Chen, H. Zhu, F. Zhang et al., Synthesis of ZIF-L-derived hollow carbons for zinc-ion capacitors. ACS Sustainable Chem. Eng. **11**(9), 3702–3709 (2023). <https://doi.org/10.1021/acssuschemeng.2c06671>
8. W. Zhang, H. Kang, Z. Gu, H. Liu, Z. Li et al., Hierarchical porous N-doped functionalized reduced graphene oxide by 2-aminoanthraquinone for aqueous zinc-ion hybrid capacitors with high energy density and ultralong-life. J. Energy Storage **61**, 106715 (2023). <https://doi.org/10.1016/j.est.2023.106715>
9. Y. Zhang, P. Xie, C. Jiang, Z. Zou, Nitrogen and oxygen Co-doped carbon micro-foams derived from gelatin as high-performance cathode materials of Zn-ion capacitors. J. Energy Storage **57**, 106169 (2023). <https://doi.org/10.1016/j.est.2022.106169>
10. F. Wei, H. Zhang, X. Hui, Y. Lv, S. Ran et al., N doped porous carbon nanosheets with enhanced zinc ion storage capability. J. Power Sources **554**, 232348 (2023). <https://doi.org/10.1016/j.jpowsour.2022.232348>
